# Wavelength-Specific Reprogramming of the Extracellular Metabolome in Bacterial CdS Biohybrids

**DOI:** 10.3390/microorganisms14102218

**Published:** 2026-10-02

**Authors:** Tan Ke, Xiaojie Ni, Liang Guo, Kai Zheng, Keren Lu, Yue Tao

**Affiliations:** 1 Zhejiang International Joint Laboratory on Low-Carbon Pollution Control and Resource Utilization, School of Biological and Environmental Engineering, Zhejiang Shuren University, Hangzhou 310015, China; hni240131@gmail.com (X.N.); liangguo93@126.com (L.G.); zhengkai9822@gmail.com (K.Z.); lukeren@njust.edu.cn (K.L.); 2 State Key Laboratory of Vegetation Structure, Function and Construction (VegLab), Institute of Ecology, College of Urban and Environmental Sciences, Peking University, Beijing 100080, China; taoyue@whu.edu.cn

**Keywords:** cadmium remediation, bacteria–semiconductor biohybrids, extracellular metabolomics, CdS nanoparticles, wavelength-dependent regulation

## Abstract

Microorganism–semiconductor biohybrids integrate biological metabolism with inorganic light-harvesting materials to enable solar-driven chemical transformations; however, how incident light wavelength regulates extracellular metabolic states remains largely unexplored. Unlike previous hybrid systems that have generally employed fixed-wavelength illumination and bacteria without plant-growth-promoting properties, we investigated wavelength-dependent reprogramming of extracellular metabolism in a CdS biohybrid based on the plant-growth-promoting bacterium *Priestia* sp. ZJT-3. Sucrose supported the highest bacterial growth, while L-cysteine alleviated Cd^2+^ stress through H_2_S-mediated CdS biomineralization, with maximal Cd removal at a cysteine/Cd^2+^ molar ratio of 2:1. Morphological analyses confirmed the formation of surface-associated CdS nanoparticles with an average size of 6.41 nm and a cubic crystal structure. Wavelength-dependent illumination markedly altered the extracellular metabolome, with the number of upregulated metabolites relative to the dark control decreasing in the order of 400, 450, and 520 nm. This pattern suggests that wavelength-dependent CdS excitation modulates photochemical interactions at the biohybrid interface and is associated with distinct bacterial metabolic responses. The 450 nm treatment produced a comparatively moderate metabolic response and was associated with increased levels of plant growth-promoting hormones, whereas 400 nm induced the strongest metabolic remodeling but also greater CdS photocorrosion and cellular morphological perturbation. These findings demonstrate that illumination wavelength can serve as a tunable parameter for regulating extracellular metabolism in bacteria–semiconductor biohybrids, highlighting the potential of such biohybrids for practical applications in areas such as light-controlled metabolite production and bioengineering.

## 1. Introduction

Microbial–semiconductor biohybrids combine the metabolic versatility of microorganisms with the light-harvesting capabilities of semiconductors, providing a promising platform for solar-to-chemical conversion [1,2]. Recent studies have shown that light-driven biohybrid systems can enhance CO_2_ conversion and contaminated-water remediation compared with microbial or nanomaterial systems alone [3,4]. Owing to their high catalytic selectivity, mild operating conditions, low energy requirements, and environmental compatibility, microbial biohybrids have been developed as emerging platforms for sustainable biomanufacturing of valuable chemicals, including acetate, shikimic acid, and bioplastics [5].

With the advancement of omics technologies, biohybrid research has increasingly shifted from performance evaluation toward understanding molecular mechanisms and metabolic functions. Semiconductor–microbe biohybrids exhibit differential regulation of genes and pathways involved in membrane transport, redox metabolism, energy metabolism, and electron transfer. For example, in *Yarrowia lipolytica*-cadmium sulfide (CdS) biohybrids, electron transfer-related functions were significantly altered, with enrichment of pathways associated with flavin adenine dinucleotide binding, monooxygenase, and oxidoreductase activities [6]. In *Clostridium ljungdahlii*-CdS biohybrids, photoinduced electrons increased the NADH/NAD^+^ ratio and electron transfer rate, indicating that semiconductor-derived electrons can alter microbial redox metabolism [7]. Engineered biosynthetic pathways can further expand the product spectrum of light-driven biohybrids, enabling the production of value-added chemicals such as 2,3-butanediol from wastewater feedstocks [8]. Collectively, these studies demonstrate that semiconductor-driven electron transfer can reshape microbial metabolism and provide a mechanistic basis for linking light input to metabolic outputs.

As a light-harvesting platform, microbial–semiconductor biohybrids are intrinsically coupled with optical parameters during solar-to-chemical energy conversion. Previous studies have demonstrated that illumination conditions, particularly light intensity, substantially influence biohybrid performance and cellular metabolism. Under absorbable wavelengths, the metabolic activity of biohybrid systems exhibited a light-intensity-dependent response, as the acetic acid production rate of the *Moorella thermoacetica*-CdS biohybrid increased with increasing blue light intensity (435–485 nm) [9]. However, excessive photon input does not necessarily improve biohybrid performance and may instead increase oxidative stress [10,11]. The wavelength of incident light can also markedly influence the performance of semiconductor–microbe biohybrids. CdS quantum dots significantly enhanced methane production by *Rhodopseudomonas* cells under 400–500 nm irradiation, whereas only marginal or no improvement was observed above 500 nm, consistent with the stronger CdS light absorption at shorter wavelengths [12]. In an *Escherichia coli*-CdS system, the apparent quantum efficiency of hydrogen production differed under 470 and 620 nm irradiation, reaching 7.93% and 9.59%, respectively, further demonstrating the influence of incident wavelength on biohybrid photochemical performance [13]. However, existing studies have primarily evaluated wavelength effects through functional outputs such as methane and hydrogen production, while the corresponding metabolic responses of microbial cells remain poorly understood. Characterizing wavelength-dependent changes in extracellular metabolites may therefore provide insight into how light wavelength reshapes microbial metabolism and regulates the functional state of semiconductor–microbe biohybrids.

CdS is one of the most widely investigated photocatalytic semiconductors, and biosynthesized CdS nanoparticles exhibit strong visible-light absorption [14]. However, commonly used bacterial hosts for CdS-based biohybrids, such as *M. thermoacetica* and *C. ljungdahlii*, generally exhibit relatively slow growth, which may limit their integration with plant-associated remediation systems. Fast-growing, plant-growth-promoting bacteria may provide a more compatible platform for such applications, particularly by utilizing organic substrates available in plant-associated environments. Here, we developed a bacteria–CdS biohybrid system based on bacterial cysteine metabolism-mediated H_2_S generation for in situ CdS biomineralization. A plant-growth-promoting and heavy metal-resistant *Priestia* sp. ZJT-3 was selected as the microbial chassis. Isolated from a heavy-metal-contaminated mining area, this bacterium grows rapidly under oxic conditions and it showed the ability to secrete the plant hormone indole-3-acetic acid (IAA) in our preliminary experiments. We hypothesized that wavelength-dependent CdS photoexcitation could differentially modulate photogenerated charge transfer and bacterial redox and energy metabolism, thereby reshaping extracellular metabolite production and secretion. We first established the optimal conditions for bacterial growth and Cd removal, and further characterized biologically synthesized CdS nanoparticles. Subsequently, extracellular metabolomics was employed to investigate wavelength-dependent metabolic remodeling of the CdS biohybrid under 400, 450, and 520 nm illumination, which were selected to represent strong, moderate, and weak CdS photoexcitation according to the wavelength-dependent absorption characteristics of CdS. By integrating semiconductor optical properties with extracellular metabolic responses, this study reveals how incident light wavelength regulates metabolite production and secretion in bacteria–semiconductor systems, providing new insights into light-mediated microbial adaptation and potential plant–biohybrid interactions.

## 2. Materials and Methods

### 2.1. Microbial Strain and Chemicals

The *Priestia* sp. ZJT-3 strain (NCBI GenBank accession number: PV688044) used in this study was isolated from a historically Cu-contaminated mining site (Tonglushan, Daye, Huangshi, Hubei Province, China) co-contaminated with Cd. All chemicals were of analytical grade and purchased from Aladdin Biochemical Technology Co., Ltd. (Shanghai, China). The H_2_S production assay kit was obtained from Grace Biotechnology Co., Ltd. (Suzhou, China) and used for quantification of bacterial H_2_S production. All solvents used for metabolomic analysis, including methanol and acetonitrile (Fisher Chemical, Waltham, MA, USA), formic acid (CNW Technologies GmbH, Düsseldorf, Germany), and 2-propanol (Merck KGaA, Darmstadt, Germany), were of High-Performance Liquid Chromatography (HPLC) grade.

### 2.2. Bacterial Growth Under Various Carbon Sources and Cd^2+^ Concentrations

Bacterial cultures were initiated by transferring cells from glycerol stocks into liquid Luria–Bertani medium, followed by overnight cultivation. Exponentially growing cells were inoculated into modified nitrogen medium at a 2% (*v*/*v*) inoculation ratio and cultivated aerobically at 37 °C with shaking at 150 rpm. The modified nitrogen medium contained (per liter): sucrose, 10.0 g; (NH_4_)_2_SO_4_, 1.0 g; K_2_HPO_4_, 0.5 g; MgSO_4_·7H_2_O, 0.5 g; NaCl, 0.1 g; and yeast extract, 0.3 g (pH 7.2). To evaluate carbon source utilization, sucrose, glucose, sodium acetate, glycerol, and mannitol were individually supplied as carbon sources, with the carbon input normalized to an equivalent amount among all treatments (0.0351 mol g^−1^). For Cd^2+^ tolerance experiments, CdCl_2_·2.5H_2_O was dissolved to prepare a Cd^2+^ stock solution and added to the culture medium to obtain final Cd^2+^ concentrations of 0, 2.5, 5, 7.5, and 10 mg L^−1^. Bacterial growth was evaluated by measuring OD_600_ at 2, 4, 8, and 24 h after inoculation. The pH of the culture supernatant was determined after 24 h of cultivation.

### 2.3. Cd^2+^ Removal and CdS Biomineralization by Cysteine-Supplemented Bacteria

To evaluate Cd^2+^ removal efficiency at different Cd^2+^ concentrations, exponentially growing cells were harvested and inoculated into modified nitrogen medium with an initial OD_600_ of 1.0. Cd^2+^ was subsequently added to final concentrations of 5, 10, 15, and 20 mg L^−1^. After 24 h cultivation, culture supernatants were collected for Cd^2+^ determination. For Cd^2+^ removal kinetics, the initial Cd^2+^ concentration was fixed at 10 mg L^−1^, and samples were collected at 3, 6, 9, 12, and 24 h after inoculation. Residual Cd^2+^ concentrations in the supernatants were quantified using inductively coupled plasma optical emission spectrometry (Agilent 5110; Agilent Technologies, Santa Clara, CA, USA).

### 2.4. Effect of Cysteine Availability on Cd^2+^ Removal and CdS Biomineralization

To determine the influence of cysteine availability on Cd^2+^ removal and CdS formation, the initial Cd^2+^ concentration was maintained at 10 mg L^−1^, while cysteine was supplied at different cysteine/Cd^2+^ molar ratios (0.5:1, 1:1, 2:1, 3:1, and 4:1). After 12 h of cultivation, culture supernatants were collected, and the concentrations of H_2_S and residual Cd^2+^ were determined.

### 2.5. Characterization of Synthesized Biohybrids

After 24 h cultivation with an initial Cd^2+^ concentration of 5 mg L^−1^, bacteria–CdS biohybrid samples were harvested by centrifugation for morphological and structural characterization. For scanning electron microscope (SEM) analysis, collected cells were fixed with 2.5% glutaraldehyde overnight and dehydrated through a graded ethanol series (10%, 30%, 50%, 70%, 90%, and 100%). The dehydrated samples were subsequently examined using a field-emission SEM (ZEISS Sigma 360; Carl Zeiss AG, Oberkochen, Germany). For transmission electron microscope (TEM) analysis, fixed samples were washed three times with phosphate-buffered saline (pH 7.4), followed by postfixation with 1% osmium tetroxide (OsO_4_) for 1–2 h. Samples were dehydrated using a graded ethanol series (30%, 50%, 70%, 90%, and 100%), embedded in resin, and sectioned into ultrathin slices (70–90 nm) using an ultramicrotome. The sections were mounted onto copper grids, stained, and examined using a TEM (Hitachi HT-7800; Hitachi High-Tech Corporation, Tokyo, Japan).

### 2.6. Extraction of CdS Nanoparticles

CdS nanoparticles were extracted following a previously reported protocol with minor modifications [15]. After 24 h of biohybrid cultivation, cells were harvested by centrifugation and resuspended in Tris-HCl buffer (50 mM, pH 7.5). Cell disruption was performed by probe sonication in an ice bath for 3 h. The resulting lysate was centrifuged at 13,000 rpm for 1 h, and the CdS nanoparticle-enriched fraction was collected. The recovered nanoparticles were dispersed in a Li_2_S/formamide solution (5 g L^−1^) and sonicated for 15 min. Subsequently, a toluene solution containing didodecyldimethylammonium bromide (DDAB, 50 mg mL^−1^) was added, followed by overnight stirring to promote nanoparticle phase transfer. The nanoparticles transferred into the upper organic phase were collected and sequentially washed with acetone and ethanol to remove residual DDAB. The purified nanoparticles were finally collected by centrifugation (10,000 rpm, 10 min) and redispersed in toluene for subsequent characterization.

### 2.7. Characterization of Extracted CdS Nanoparticles

Purified nanoparticle suspensions were drop-cast onto carbon-coated copper grids and dried under ambient conditions prior to microscopic characterization. TEM imaging was performed using a JEOL JEM-F200 microscope (JEOL Ltd., Tokyo, Japan). High-angle annular dark-field scanning TEM imaging and selected area electron diffraction (SAED) analyses were conducted at an accelerating voltage of 200 kV to characterize nanoparticle morphology and crystal structure.

### 2.8. Wavelength-Dependent Regulation of Extracellular Metabolites in Bacteria–CdS Biohybrids

Bacteria–CdS biohybrids were collected and resuspended in modified nitrogen medium supplemented with L-tryptophan (200 mg L^−1^) at an initial OD_600_ of 1.0. The biohybrids were illuminated with different wavelengths (400, 450, and 520 nm) at an irradiance of 2.0 mW cm^−2^ under shaking conditions (150 rpm) for 16 h. Subsequently, cell density, morphology, and supernatant Cd^2+^ concentrations were determined.

For extracellular metabolite extraction, 100 μL of culture supernatant was mixed with 400 μL of precooled acetonitrile/methanol (1:1, *v*/*v*) containing internal standards. The samples were vortexed for 30 s, sonicated at 5 °C for 30 min, and incubated at −20 °C for 30 min to precipitate proteins. After centrifugation at 13,000× *g* for 15 min at 4 °C, the supernatants were collected, evaporated to dryness under nitrogen, and reconstituted in 100 μL acetonitrile/water (1:1, *v*/*v*). Following sonication for 5 min at 5 °C and centrifugation at 13,000× *g* for 10 min at 4 °C, the resulting extracts were subjected to liquid chromatography-mass spectrometry (LC-MS/MS) analysis.

Metabolomic profiling was performed using a UHPLC system coupled with an Orbitrap Exploris 480 mass spectrometer (Thermo Fisher Scientific, Waltham, MA, USA) equipped with an ACQUITY HSS T3 column (100 mm × 2.1 mm, 1.8 μm; Waters Technologies Corporation, Milford, MA, USA). Chromatographic separation was performed at 40 °C with a flow rate of 0.40 mL min^−1^ and an injection volume of 3 μL. The mobile phases consisted of water/acetonitrile (95:5, *v*/*v*) containing 0.1% formic acid and acetonitrile/isopropanol/water (47.5:47.5:5, *v*/*v*/*v*) containing 0.1% formic acid. Mass spectrometry data were acquired in both positive and negative electrospray ionization modes using data-dependent acquisition over an *m*/*z* range of 70–1050 with stepped collision energies of 20, 40, and 60 V.

Raw MS data were processed using Progenesis QI (v3.0) for peak detection, retention time alignment, and metabolite annotation by matching against public databases and the Majorbio in-house database (Majorbio Bio-Pharm Technology Co., Ltd., Shanghai, China). A pooled quality control (QC) sample generated by mixing equal volumes of all samples was analyzed periodically to evaluate analytical stability. Metabolic features detected in ≥80% of samples were retained, followed by missing-value imputation, total ion normalization, QC-based filtering (relative standard deviation ≤ 30%), and log_10_ transformation. Principal component analysis (PCA) and orthogonal partial least squares discriminant analysis (OPLS-DA) were performed using the ropls package (v1.6.2) in R. Metabolites with variable importance in projection (VIP) values > 1 and *p* < 0.05 were considered significantly altered and subsequently subjected to Kyoto Encyclopedia of Genes and Genomes (KEGG) pathway enrichment analysis.

### 2.9. Statistical Analysis

Metabolomic analyses were performed using four biological replicates, whereas all other experiments were conducted with three biological replicates. Data are presented as mean ± standard error of the mean. Figures were generated using Origin 2024 and the ggplot2 package (v4.0.1) implemented in R (v4.5.2). The significance of differences among multiple experimental groups was assessed using one-way analysis of variance in SPSS (version 26.0), followed by Duncan’s multiple range test for pairwise comparisons. Differences were considered statistically significant when *p* < 0.05.

## 3. Results and Discussions

### 3.1. The Response of Bacterial Growth to Carbon Sources and Cd^2+^

**Figure 1:**
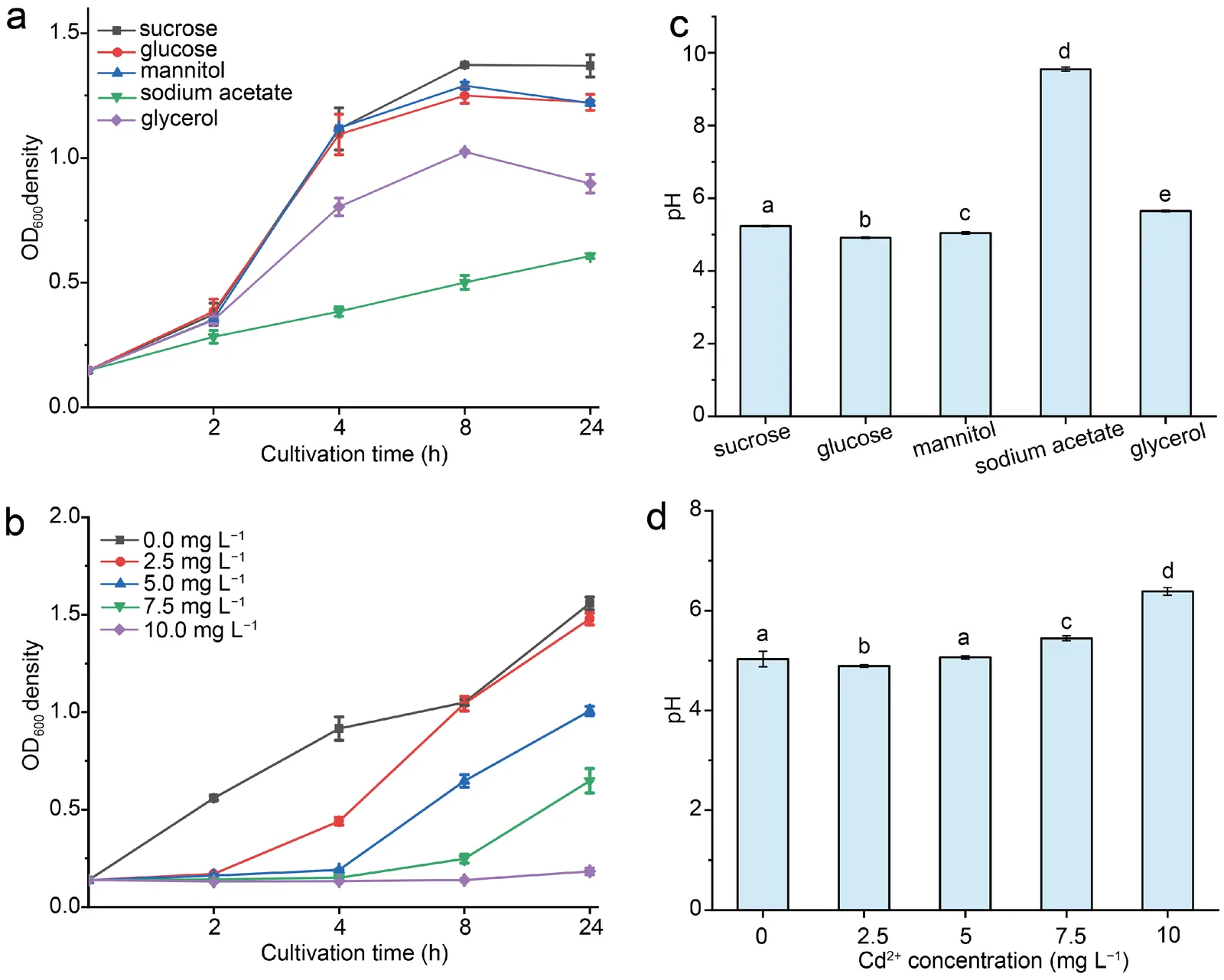
Growth and pH responses of *Priestia* sp. ZJT-3 under different carbon sources and Cd^2+^ concentrations. (**a**,**b**), OD_600_ values of *Priestia* sp. ZJT-3 cultivated with different carbon sources (**a**) and exposed to various Cd^2+^ concentrations (**b**). (**c**,**d**), Changes in culture pH of *Priestia* sp. ZJT-3 under different carbon sources (**c**) and Cd^2+^ concentrations (**d**). Different lowercase letters (a–e) above the bars indicate statistically significant differences among the groups (*p* < 0.05), whereas bars sharing the same letter are not significantly different.

As shown in Figure 1a, *Priestia* ZJT-3 exhibited distinct growth patterns when supplied with different carbon sources. After 24 h cultivation, the highest biomass accumulation was observed in the sucrose group (OD_600_ = 1.370 ± 0.045), whereas sodium acetate supported the lowest growth (OD_600_ = 0.607 ± 0.010). During the initial 4 h cultivation, *Priestia* ZJT-3 showed comparable growth trends in the glucose, mannitol, and sucrose groups; however, sucrose subsequently promoted faster biomass accumulation, resulting in higher cell density after 4 h. Cd^2+^ exposure markedly inhibited bacterial growth in a concentration-dependent manner (Figure 1b). After 24 h cultivation, the highest OD_600_ value was observed at 2.5 mg L^−1^ Cd^2+^ (1.479 ± 0.030), which was comparable to the Cd^2+^-free control (1.550 ± 0.033), whereas 10 mg L^−1^ Cd^2+^ substantially suppressed bacterial growth (OD_600_ = 0.183 ± 0.017). Changes in culture pH further reflected distinct metabolic responses under different carbon sources and Cd^2+^ stress conditions (Figure 1c,d). After 24 h cultivation, the supernatant pH ranged from 4.92 ± 0.02 to 5.65 ± 0.03 in most carbon source treatments, whereas the sodium acetate group exhibited an alkaline environment (pH 9.55 ± 0.02), likely due to acetate metabolism and the absence of buffering components in the modified medium. Notably, although the sucrose group showed the highest biomass production, its final pH was not the lowest, indicating that different carbon sources resulted in distinct patterns of organic acid production. Under Cd^2+^ stress, treatments with relatively higher bacterial growth generally exhibited lower pH values, suggesting a close association between bacterial growth, metabolic activity, and culture acidification.

Bacterial growth is highly dependent on carbon source availability and metabolic compatibility. Diverse carbon sources, including carbohydrates, fatty acids, amino acids, and aromatic compounds, can be utilized by microorganisms to support cellular growth. Previous studies reported that the maximum specific growth rate of bacteria was higher with sucrose (0.544 h^−1^) than with glucose (0.526 h^−1^) or glycerol (0.408 h^−1^) [16]. And our observation of limited biomass production with glycerol and sodium acetate agrees with previous findings that *Lactobacillus* exhibited lower biomass accumulation under glycerol, with OD_595_ remaining below 0.5 after 24 h cultivation, whereas glucose supported an OD_595_ of approximately 1.5 [17]. The decrease in culture pH was mainly attributed to the production of organic acids during bacterial carbon metabolism. Organic acids produced during microbial carbon metabolism can influence metal mobility and availability [18,19]. Bacterial isolates have been reported to secrete organic acids, including oxalic acid, acetic acid, and citric acid, as well as biosurfactants, thereby promoting metal mobilization in soil environments [20].

Cd is a widespread environmental contaminant and a nonessential metal that poses potential risks to biological systems and human health. Even at relatively low concentrations, Cd can exert toxic effects by disrupting essential cellular processes, including membrane integrity, protein function, and metabolic activity [21,22]. In bacteria, Cd^2+^ exposure has been shown to prolong lag phases, decrease growth rates, impair carbon assimilation, and ultimately reduce microbial biomass [23,24]. Moreover, Cd-induced oxidative stress can lead to cellular damage and enzyme inactivation, with Cd^2+^ exhibiting stronger toxicity toward bacterial cells than other heavy metals such as Cu^2+^ [25]. Long-term accumulation of Cd and other heavy metals in soils can further disrupt microbial communities by reducing the activities of key soil enzymes, including acid phosphatase and urease [22]. Consistent with previous reports on plant growth-promoting bacteria [26,27], Cd^2+^ also exhibited inhibitory effects on the growth of *Priestia* ZJT-3 in this study.

### 3.2. Bioremoval of Cd^2+^ and Biomineralization of CdS by Priestia ZJT-3

**Figure 2:**
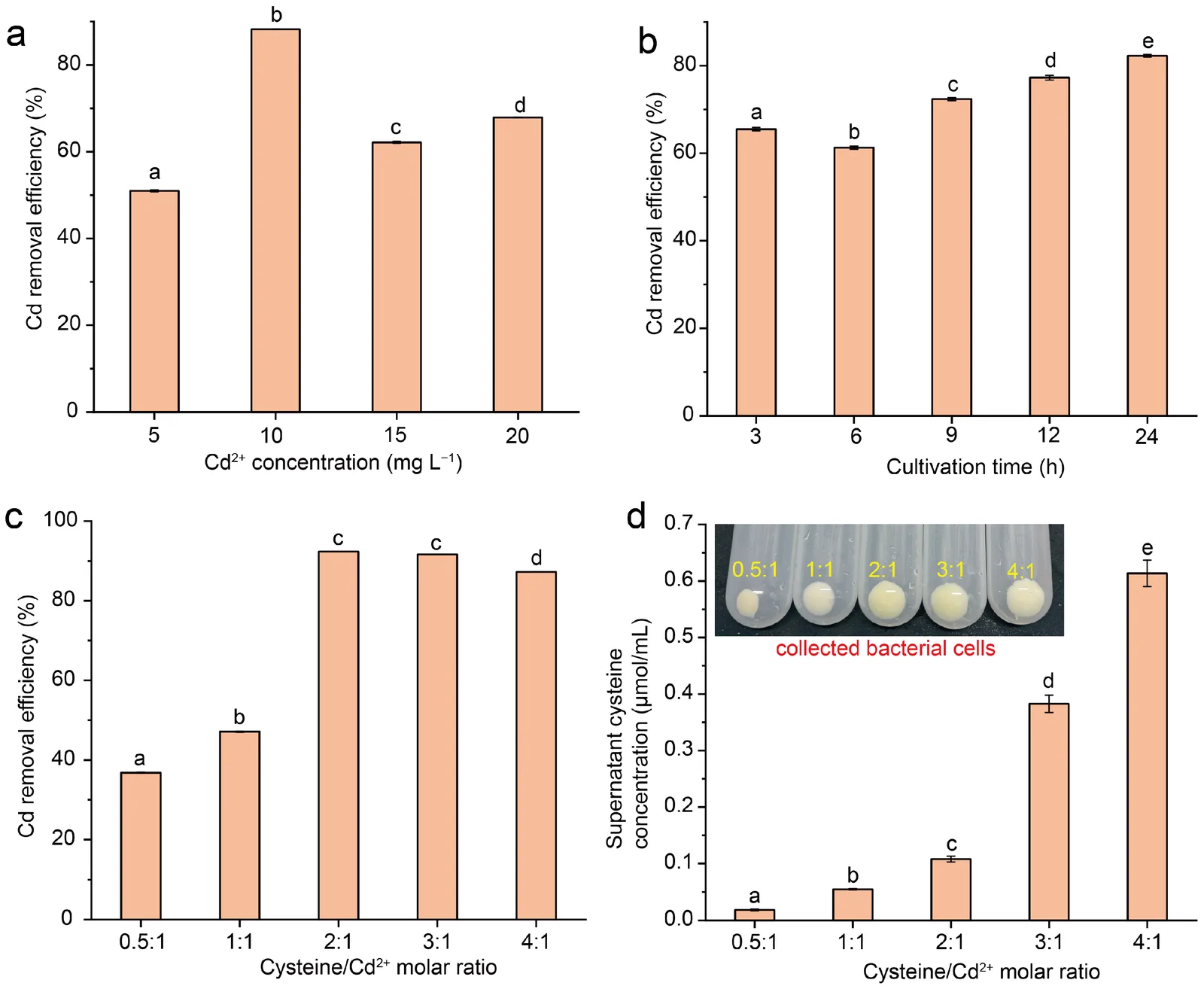
Cysteine-mediated Cd^2+^ removal and H_2_S production by *Priestia* ZJT-3. (**a**), Cd^2+^ removal efficiency of *Priestia* ZJT-3 after 24 h cultivation with initial Cd^2+^ concentrations of 5, 10, 15, and 20 mg L^−1^. (**b**), Time-dependent Cd^2+^ removal efficiency of *Priestia* ZJT-3 during 3, 6, 9, 12, and 24 h cultivation with an initial Cd^2+^ concentration of 10 mg L^−1^. (**c**), Effect of cysteine/Cd^2+^ molar ratios (0.5:1, 1:1, 2:1, 3:1, and 4:1) on Cd^2+^ removal efficiency after 12 h cultivation with an initial Cd^2+^ concentration of 10 mg L^−1^. (**d**), H_2_S concentration in culture supernatants under different cysteine/Cd^2+^ molar ratios after 12 h cultivation. Different lowercase letters (a–e) above the bars indicate statistically significant differences among the groups (*p* < 0.05), whereas bars sharing the same letter are not significantly different.

Cysteine plays a dual role in microbial metal stress responses by improving cellular tolerance and providing sulfur sources for the biosynthesis of metal sulfides through H_2_S production. When exposed to 5–20 mg L^−1^ Cd^2+^, *Priestia* ZJT-3 exhibited effective Cd^2+^ removal, with an average removal efficiency of 67.32%, and the highest removal efficiency (88.24 ± 0.08%) was achieved at 10 mg L^−1^ Cd^2+^ (Figure 2a). Cd^2+^ removal progressively increased with cultivation time, increasing from 65.49 ± 0.38% after 3 h to 82.27 ± 0.29% after 24 h (Figure 2b), indicating a time-dependent Cd^2+^ sequestration process. The influence of cysteine availability on Cd^2+^ removal was further investigated by adjusting the cysteine/Cd^2+^ molar ratio. After 12 h cultivation, Cd^2+^ removal efficiencies ranged from 36.80 ± 0.10% to 92.36 ± 0.02% under different cysteine/Cd^2+^ ratios (Figure 2c). The highest removal efficiency was obtained at a cysteine/Cd^2+^ ratio of 2:1, whereas insufficient cysteine supply (0.5:1) resulted in the lowest removal efficiency. The H_2_S concentration in the culture supernatant increased from 0.019 ± 0.002 to 0.614 ± 0.024 μmol L^−1^ with increasing cysteine availability (Figure 2d), suggesting that cysteine-derived H_2_S production contributed to Cd^2+^ immobilization.

Many studies have explored the application of metal-tolerant bacterial strains for heavy metal removal from water, soil, and sediments through various sorption mechanisms, including intracellular metal sequestration, adsorption onto cell surfaces, and extracellular precipitation [21,28]. However, adsorbed metal ions may be released again due to environmental fluctuations, such as changes in pH or bacterial cell lysis [29]. In contrast, biomineralization represents an effective microbial strategy for long-term metal immobilization and provides a promising approach for mitigating heavy metal contamination. Microorganisms can utilize cysteine as a sulfur source to generate sulfide species and facilitate metal sulfide precipitation. Compared with hydroxide-based precipitation, metal sulfides are generally more favorable for immobilization due to their higher reactivity and lower leaching potential [30]. Previous studies have demonstrated that L-cysteine serves not only as a precursor for H_2_S production but also as a capping ligand that regulates CdS nanoparticle growth and size [31]. In this study, cysteine supplementation enhanced Cd^2+^ tolerance and removal capacity of *Priestia* ZJT-3. Although *Priestia* ZJT-3 showed limited tolerance toward 10 mg L^−1^ Cd^2+^ in the absence of cysteine, it achieved 67.91% Cd^2+^ removal after cysteine addition, consistent with previous observations in the deep-sea bacterium *Pseudomonas stutzeri* 273 [32]. Previously reported CdS-forming bacteria, such as *M. thermoacetica* [9] and sulfate-reducing bacteria, generally exhibit relatively slow growth and limited carbon-source utilization. *E. coli* grows rapidly but lacks the plant-growth-promoting properties relevant to plant–biohybrid applications [15]. In contrast, *Priestia* sp. ZJT-3 combines rapid aerobic growth, broad carbon-source utilization, and plant-growth-promoting potential, making it a promising microbial chassis for CdS biohybrids and their potential integration with plant-based remediation systems.

### 3.3. Characterization of Bacteria–CdS Biohybrids

**Figure 3:**
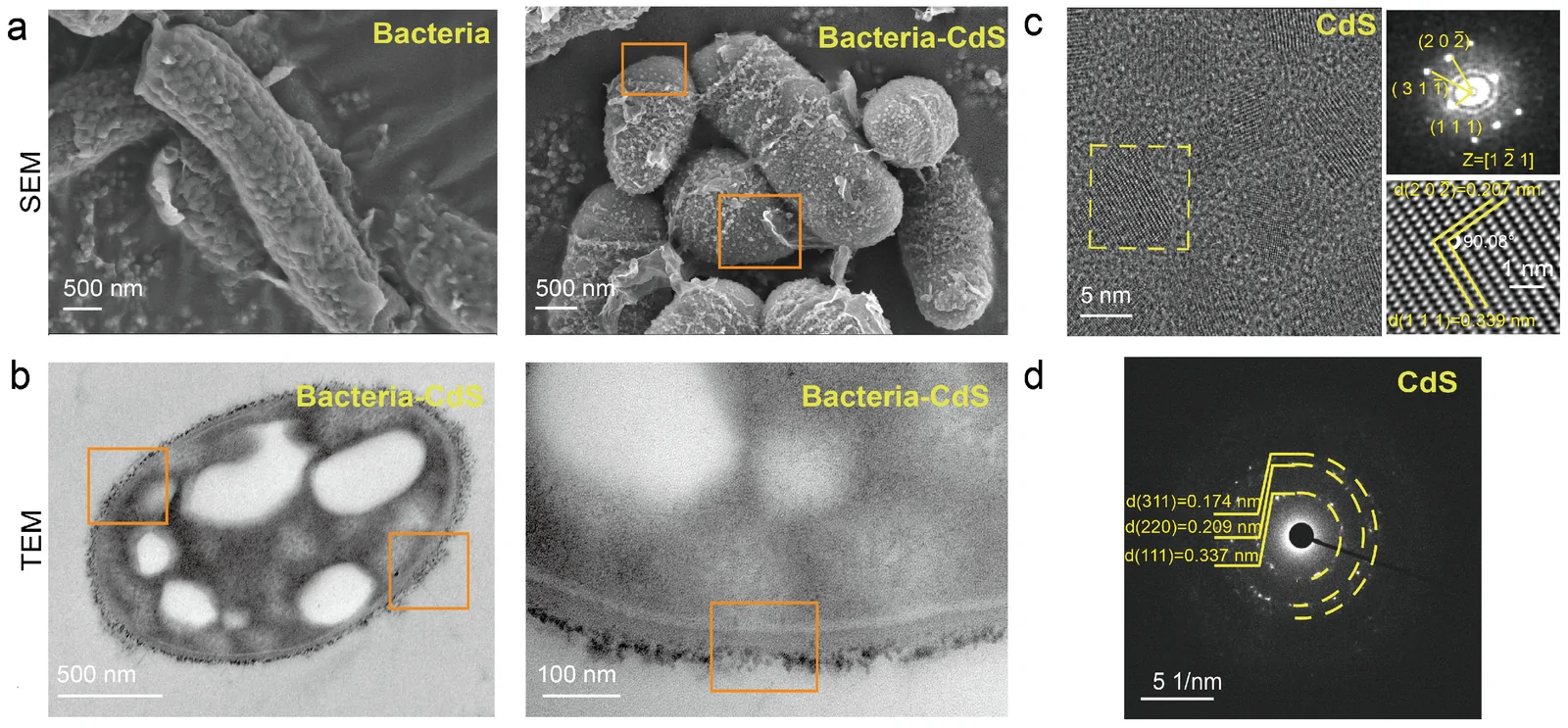
Morphological and structural characterization of CdS nanoparticles synthesized by *Priestia* ZJT-3. (**a**), SEM images of *Priestia* ZJT-3 cells and CdS-associated *Priestia* ZJT-3 biohybrids. (**b**), TEM image of the bacteria–CdS biohybrid showing surface-associated nanoparticles. (**c**), High-resolution TEM image and corresponding fast Fourier transform pattern of extracted CdS nanoparticles. (**d**), SAED pattern of extracted CdS nanoparticles. Orange boxes indicate bacterial-synthesized CdS nanoparticles; the yellow dashed box marks the crystalline region selected for fast Fourier transform analysis; the angle marker denotes the angle between various planes; and dashed arcs in the SAED pattern highlight various diffraction rings.

SEM imaging revealed distinct nanoparticle-like structures attached to the bacterial surface when Cd^2+^ and L-cysteine were simultaneously supplied, whereas no comparable structures were observed in the Cd^2+^-free control (Figure 3a). The surface-associated particles exhibited sizes ranging from 18.95 to 61.95 nm, with an average diameter of 34.29 nm (Figure S1). Consistent with SEM observations, TEM analysis confirmed that the yellow-colored nanoparticles were predominantly localized on the outer surface of bacterial cells (Figure 3b), indicating the formation of a bacteria-associated CdS biohybrid.

To further characterize the intrinsic properties of the semiconductor nanoparticles, CdS nanoparticles were extracted from the biohybrid system and subjected to TEM analysis. Compared with the cell-associated particles, the extracted nanoparticles exhibited significantly smaller dimensions, which was likely due to the aggregation of individual CdS quantum dots on the bacterial surface. The isolated nanoparticles displayed mainly triangular morphologies with sizes ranging from 2.48 to 12.52 nm and an average diameter of 6.41 nm (Figure S2). High-resolution TEM analysis revealed well-defined lattice fringes, demonstrating the high crystallinity of the extracted nanoparticles (Figure 3c). Furthermore, the SAED pattern showed three characteristic diffraction rings with interplanar spacings of 0.337, 0.209, and 0.174 nm, corresponding to the (111), (220), and (311) planes of cubic CdS, respectively (Figure 3d).

Microbial synthesis of CdS nanoparticles has been reported in diverse microorganisms, including bacteria, fungi, and yeasts, through intracellular or extracellular sulfur metabolism pathways [33]. Biologically synthesized CdS nanoparticles typically exhibit nanoscale dimensions ranging from several to tens of nanometers. For example, *M. thermoacetica* produced CdS nanoparticles smaller than 10 nm in the presence of Cd^2+^ and cysteine [9], whereas *Rhodopseudomonas palustris* TN110 generated spherical CdS nanoparticles with an average diameter of 4.85 nm [34]. Unlike inorganic sulfide precursors that directly provide sulfide ions, sulfur-containing amino acids such as cysteine can act as alternative sulfur donors under aerobic conditions through enzymatic H_2_S production [35]. Previous studies have shown that cysteine-derived H_2_S reacts with Cd^2+^ in intracellular or periplasmic regions, leading to the formation of CdS nanoclusters in *E. coli* [15]. In this study, CdS nanoparticles were mainly localized on the bacterial surface, indicating that cysteine-mediated sulfide generation promoted surface-associated CdS biomineralization in *Priestia* ZJT-3. The efficiency of CdS formation and nanoparticle characteristics can be strongly influenced by bacterial growth stage and precursor availability [36], which may explain the variation in Cd removal efficiency observed under different cysteine/Cd^2+^ molar ratios. The resulting bacteria–CdS biohybrid systems integrate the metabolic versatility of microorganisms with the photoactive properties of semiconductors, enabling light-induced electron transfer and subsequent chemical transformations, including CO_2_ reduction and organic compound conversion into value-added products. Recently, an engineered *Vibrio natriegens*–CdS biohybrid system was scaled up to 50 L using wastewater as the feedstock, achieving over 99% heavy metal removal and enhanced 2,3-butanediol production (12 g L^−1^), demonstrating the potential of microbial semiconductor hybrids for practical applications [37].

### 3.4. Wavelength-Dependent Extracellular Metabolic Reprogramming in Bacteria–CdS Biohybrids

**Figure 4:**
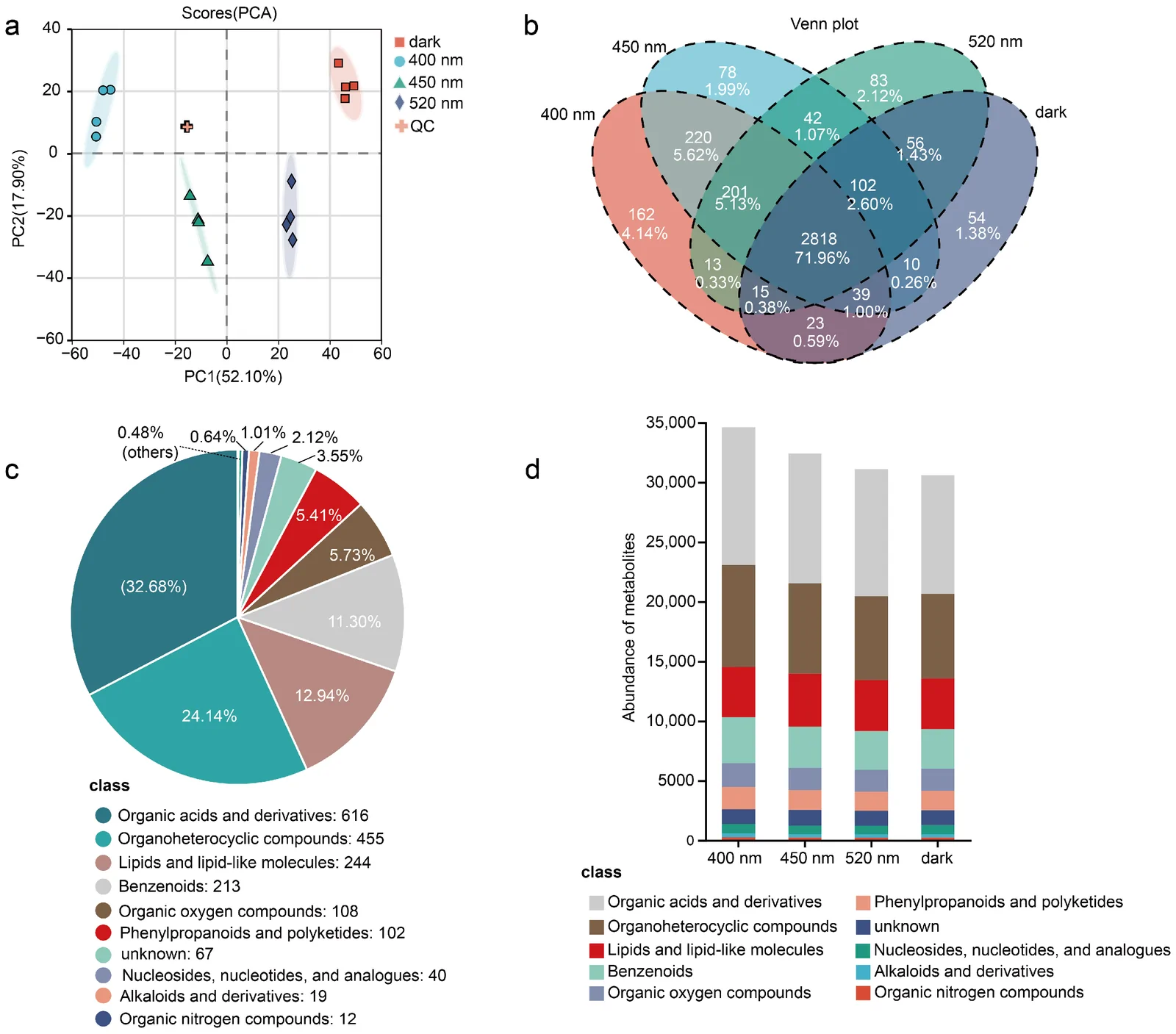
Extracellular metabolomic profiling of *Priestia* ZJT-3-CdS biohybrids under dark conditions and different wavelengths of illumination. (**a**), PCA of extracellular metabolite profiles among different treatment groups. (**b**), Venn diagram showing shared and wavelength-specific metabolites among different groups. (**c**), Distribution of identified metabolites according to chemical classes. (**d**), Stacked bar chart showing the abundance of metabolites belonging to different chemical classes across different treatments.

To investigate whether different wavelengths modulated extracellular metabolism, *Priestia* ZJT-3-CdS biohybrids were cultivated under dark conditions and illuminated with 400, 450, and 520 nm light. Untargeted metabolomic analysis annotated 3311 metabolites through database matching. Clear separation among wavelength-treated and dark groups was observed, indicating pronounced wavelength-dependent metabolic remodeling under photoexcitation (Figure 4a and Figure S3). PCA result demonstrated distinct metabolic profiles among the different treatments, with PC1 and PC2 explaining 52.1% and 17.9% of the total variance, respectively, accounting for approximately 70% of the overall variation. Biological replicates within each treatment clustered closely in the PCA, indicating high reproducibility and low within-group variation. The Venn diagram analysis revealed wavelength-specific metabolite signatures, with 162, 78, 83, and 54 unique metabolites detected in the 400, 450, 520 nm, and dark groups, respectively (Figure 4b). Chemical classification analysis showed that the identified metabolites were primarily assigned to organic acids and derivatives (616 metabolites), organoheterocyclic compounds (455 metabolites), lipids and lipid-like molecules (244 metabolites), benzenoids (213 metabolites), organic oxygen compounds (108 metabolites), and phenylpropanoids and polyketides (102 metabolites) (Figure 4c). Organic acids and derivatives represented the predominant metabolite class across all treatments, with the highest abundance observed under 400 nm illumination and the lowest abundance in the dark condition (Figure 4d). Organoheterocyclic compounds exhibited a wavelength-dependent decline, decreasing from 8557.18 under 400 nm illumination to 7038.91 under 520 nm illumination. In contrast, lipids and lipid-like molecules remained relatively stable across different illumination conditions. Moreover, aromatic metabolite classes, including benzenoids and phenylpropanoids/polyketides, showed reduced abundance under longer-wavelength irradiation, particularly in the 520 nm group. Together, these results demonstrate that illumination wavelength serves as an important regulatory parameter for shaping extracellular metabolite composition in bacterial CdS biohybrid systems.

**Figure 5:**
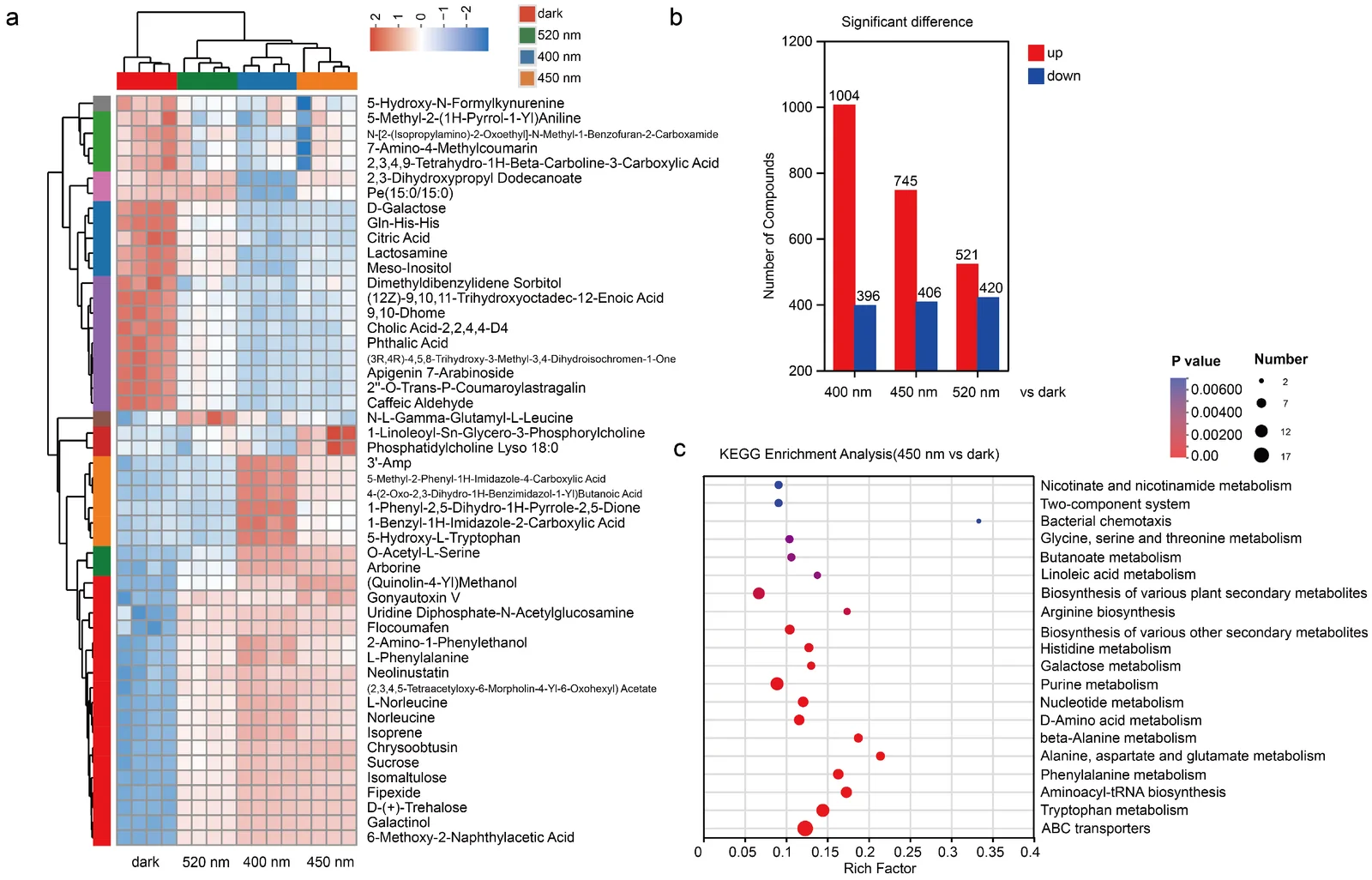
Wavelength-dependent remodeling of extracellular metabolites in *Priestia* ZJT-3-CdS biohybrids. (**a**), Hierarchical clustering heatmap showing wavelength-dependent patterns of extracellular metabolites among different treatment groups. (**b**), Differentially altered extracellular metabolites in biohybrids under different wavelengths of illumination compared with the dark control. (**c**), KEGG pathway enrichment analysis of differential extracellular metabolites in 450 nm-illuminated biohybrids compared with the dark control.

Hierarchical clustering and PLS-DA score plots revealed distinct wavelength-dependent remodeling of extracellular metabolites in *Priestia* ZJT-3-CdS biohybrids (Figure 5a and Figures S4 and S5). Notably, the metabolite profiles under 520 nm illumination clustered closely with the dark control, consistent with the limited CdS excitation efficiency at this wavelength due to its proximity to the CdS absorption edge (~424 nm) [30]. These results suggest that insufficient photon absorption by CdS resulted in minimal perturbation of extracellular metabolic composition. In contrast, 400 and 450 nm illumination induced pronounced deviations from the dark condition. Compared with the dark control, 1004, 745, and 521 significantly altered metabolites were detected under 400, 450, and 520 nm illumination, respectively (Figure 5b and Figures S6–S8). The number of differential metabolites decreased progressively with increasing wavelength from 400 to 520 nm, indicating weaker metabolic perturbation at longer wavelengths.

Analysis of the top 30 VIP-ranked differential metabolites further revealed wavelength-specific metabolic signatures. At 400 nm, the differential metabolites were dominated by aromatic, sulfur-, and amino acid-related compounds, accompanied by greater cellular stress, whereas 450 nm elicited a more coordinated peptide and amino acid response and 520 nm induced relatively limited metabolic changes. Under 400 nm irradiation, highly responsive metabolites were mainly composed of diverse aromatic and heterocyclic compounds, including Ceplignan (VIP = 3.05, fold change [FC] = 326.15), Dihydrocubebin (VIP = 2.97, FC = 50.59), Flunoxaprofen (VIP = 2.89, FC = 11.10), and 5,6-Diaminonaphthalene-1-sulfonic acid (VIP = 2.68, FC = 61.76) (Figure S9). These metabolites were primarily associated with aromatic compound and secondary metabolism, with potential links to cellular redox processes. The accumulation of structurally diverse metabolites indicated extensive remodeling of extracellular chemical composition under strong CdS photoexcitation. Meanwhile, sulfur- and amino acid-related metabolites, such as γ-Glu-Met (VIP = 2.67, FC = 3.41) and short peptides, were also increased. γ-Glu-Met is a γ-glutamyl peptide involved in bacterial amino acid homeostasis and sulfur-containing amino acid metabolism. The overall increase in these metabolites suggests enhanced peptide and sulfur metabolism, potentially reflecting adaptations in redox homeostasis and cellular stress responses under photoinduced stress. Previous studies have shown that strong CdS excitation may induce membrane damage and oxidative stress in microbial-CdS systems [9]. Consistently, prolonged 400 nm illumination in our study resulted in visible darkening of bacterial cells, further suggesting severe photoinduced stress under excessive CdS excitation. Notably, the biohybrid exposed to 400 nm irradiation showed slightly lower cell density than the other treatments (Figure S10), accompanied by apparent cell shrinkage and deformation (Figure S11). These observations suggest that some extracellular metabolites detected at 400 nm may have originated from cell lysis. The observed cellular damage may have been associated with both the high-energy 400 nm irradiation and Cd^2+^ release resulting from CdS photodissolution (Figure S12).

Compared with 400 nm irradiation, 450 nm illumination induced a more coordinated extracellular metabolic response. Although several aromatic metabolites were also enriched, the VIP-ranked metabolites were characterized by increased amino acid-derived peptides, including Leu-Glu-Val (VIP = 2.86, FC = 2.79), Pro-Val-Thr (VIP = 2.85, FC = 2.57), and Gln-Met-Leu (VIP = 2.71, FC = 2.52) (Figure S13). Gln-Met-Leu is a short peptide containing glutamine and methionine, which are associated with bacterial nitrogen and sulfur-containing amino acid metabolism, respectively. The accumulation of these peptides may reflect altered peptide turnover and amino acid availability under photoinduced conditions. Moreover, the biohybrid showed greater cellular integrity and relatively weaker CdS photocorrosion under 450 nm irradiation (Figures S10–S12). Thus, 450 nm irradiation was associated with a comparatively balanced response between CdS excitation and cellular metabolic adaptation. Under 520 nm illumination, cellular integrity was largely maintained and photodamage was limited. However, fewer metabolites were upregulated relative to the dark control, suggesting that the weaker CdS excitation at 520 nm resulted in more limited metabolic activation (Figure S14).

KEGG pathway enrichment analysis further revealed distinct pathway-level responses across wavelengths. The 400 nm treatment was primarily associated with sulfur and amino acid metabolism and stress-related pathways, whereas 450 nm was enriched in pathways related to biosynthesis, transport, and environmental sensing. In contrast, 520 nm showed comparatively limited pathway enrichment, with changes largely involving basal metabolic functions. Under 400 nm irradiation, enriched pathways were mainly associated with amino acid metabolism, sulfur metabolism, secondary metabolite biosynthesis, and stress-related adaptation (Figure S15). The enrichment of cysteine and methionine metabolism, sulfur relay system, arginine biosynthesis, and multiple amino acid metabolic pathways suggested enhanced sulfur utilization, redox regulation, and metabolic reorganization under strong CdS excitation. In comparison, 450 nm irradiation induced a more balanced metabolic pattern characterized by biosynthesis, signal transduction, and molecular exchange (Figure 5c). Notably, two-component system and bacterial chemotaxis pathways were uniquely enriched under 450 nm illumination, suggesting enhanced environmental sensing and adaptive regulation. Meanwhile, the enrichment of ABC transporters, aminoacyl-tRNA biosynthesis, nucleotide metabolism, and amino acid-related pathways indicated active regulation of metabolite transport, protein synthesis, and cellular resource allocation. These results suggest that 450 nm irradiation promoted metabolic activation without inducing excessive stress responses. Under 520 nm illumination, enriched pathways were mainly associated with basal metabolic maintenance rather than extensive metabolic remodeling (Figure S16). Pathways related to phenylalanine, tyrosine and tryptophan biosynthesis, phosphotransferase system, pantothenate and CoA biosynthesis, and glutathione metabolism suggested maintenance of fundamental cellular metabolism under weak CdS excitation.

As *Priestia* ZJT-3 possesses the ability to produce indole-3-acetic acid, a phytohormone known to promote plant root development [38], we further investigated wavelength-dependent alterations in tryptophan-associated metabolites, which serve as precursors or intermediates involved in IAA biosynthesis. Overall, extracellular IAA levels increased under both 400 nm (VIP = 1.16, FC = 1.15, *p* < 0.05) (Figure S17) and 450 nm illumination (VIP = 0.81, FC = 1.06, *p* < 0.05) (Figure S18) compared with the dark control, suggesting enhanced regulation of IAA-related metabolism under CdS excitation. Specifically, decreased L-tryptophan abundance accompanied by increased accumulation of 5-hydroxy-L-tryptophan under both wavelengths indicated enhanced turnover of tryptophan-associated metabolic networks. IAA biosynthesis in bacteria can proceed through multiple tryptophan-dependent pathways, including the indole-3-acetamide (IAM), indole-3-pyruvic acid (IPA), tryptamine (TAM), indole-3-acetonitrile (IAN), and tryptophan side-chain oxidase (TSO) pathways [39,40,41]. Analysis of pathway-associated metabolites showed that intermediates related to the IAM pathway (indole-3-acetamide, VIP = 1.05, FC = 1.08, *p* < 0.05), IPA pathway (indole-3-pyruvic acid, VIP = 1.52, FC = 1.24, *p* < 0.05), and TSO pathway (indole-3-acetaldehyde, VIP = 1.68, FC = 1.21, *p* < 0.05) were increased under 450 nm illumination, whereas TAM- and IAN-associated intermediates showed no obvious accumulation. These results suggest that CdS-mediated photoexcitation may preferentially promote specific tryptophan-dependent routes contributing to IAA production. In contrast, these metabolic alterations were substantially weaker under 520 nm illumination (Figure S19), consistent with its limited influence on CdS excitation and extracellular metabolic remodeling.

Microorganism–semiconductor biohybrid systems have recently emerged as promising platforms for enhancing microbial synthesis by coupling the photoelectric properties of semiconductors with cellular metabolism. Kelvin probe force microscopy measurements showed that CdS had a higher surface potential than the *Shewanella putrefaciens* surface, while illumination induced potential changes of approximately 5.6 mV for CdS and 3.1 mV for the bacterial surface. These observations suggest the formation of an interfacial electric field that may facilitate photogenerated electron transfer from CdS toward bacterial cells [42]. In *S. oneidensis*-CdS biohybrids, photoexcited electrons generated by extracellular CdS have been shown to enter the bacterial extracellular electron transfer pathway through outer-membrane c-type cytochromes, particularly MtrC and OmcA, and subsequently interact with periplasmic redox proteins [43]. Electron transfer into bacterial cells can alter intracellular redox states and redirect metabolic flux toward target products [44]. The wavelength-dependent changes in extracellular metabolites observed in this study may primarily reflect differences in CdS light absorption and the resulting electron flow at different wavelengths. Variations in electron flow within biohybrid systems may involve electron-transfer cofactors such as thiamine pyrophosphate and consequently alter the intracellular NADH/NAD^+^ ratio [2]. The increasing NADH/NAD^+^ ratio with prolonged illumination also highlights the need for time-resolved experiments to characterize the temporal dynamics and persistence of wavelength-dependent metabolic responses. In addition, extracellular metabolite abundance reflects not only intracellular production but also transport, secretion, extracellular transformation, and, under stress conditions, potential cell damage or lysis. Further studies should elucidate the interfacial electron-transfer mechanisms in CdS-bacterial biohybrids and clarify their links to intracellular regulatory networks. Bacteria-only and cell-free CdS controls under different wavelengths, together with temperature-controlled experiments, will be important for distinguishing direct bacterial light responses from CdS-mediated photochemical and thermal effects. Moreover, a broader and more finely resolved wavelength range beyond the three wavelengths examined here would provide a more comprehensive understanding of wavelength-dependent extracellular metabolic regulation.

Unlike previous studies that have primarily focused on applications such as H_2_ production, chemical synthesis, and pollutant degradation [37,45,46], this study further examined wavelength-dependent metabolic responses, with particular emphasis on phytohormone-associated metabolites. In the present study, extracellular IAA levels increased by 14.79%, 5.74%, and 0.73% under 400, 450, and 520 nm illumination, respectively, indicating that wavelength-dependent CdS excitation influenced phytohormone-associated metabolism. Further investigations are required to clarify their ecological significance, particularly through direct evaluation of plant growth promotion and physiological responses in the presence of in situ-formed biohybrids under optimized illumination conditions. Illumination may induce CdS transformation and the release of dissolved Cd species, potentially compromising the long-term stability of Cd immobilization. Appropriate sacrificial agents may therefore be considered in future plant–biohybrid applications. The phytotoxicity of different Cd species should also be carefully evaluated, as CdS nanoparticles themselves have been reported to exert toxic effects on plants [47]. From a practical perspective, recent work demonstrated the scalability of semiconductor–microbe biohybrids to a 50 L reactor using actual nonsterile wastewater, where biohybrid formation enhanced 2,3-butanediol production [37]. The aerobic, fast-growing *Priestia* sp. ZJT-3 used here can utilize a broad range of carbon sources, potentially facilitating its cultivation and scale-up for biohybrid production. Blue light around 450 nm is readily available from sunlight and can be supplied using commercial LEDs, while also being utilized by plants for photosynthesis. Thus, 450 nm illumination may be compatible with plant–biohybrid remediation systems; however, its long-term performance under natural broadband sunlight and its effects on Cd stability and plant health require further validation.

## 4. Conclusions

In this study, a plant-growth-promoting and metal-tolerant *Priestia* sp. ZJT-3 was used to establish a CdS-based bacteria–semiconductor biohybrid and investigate how light wavelength regulates its extracellular metabolic state. The results demonstrate that H_2_S-producing bacterial metabolism can support in situ CdS biomineralization, providing a microbial route for constructing functional semiconductor–microbe interfaces. More importantly, this study identifies illumination wavelength as a controllable parameter for regulating extracellular metabolic outputs in CdS biohybrids. The results suggest that wavelength-dependent CdS excitation is associated with distinct bacterial metabolic responses, with shorter wavelengths inducing stronger metabolic remodeling but greater cellular perturbation, whereas longer wavelengths produced weaker metabolic changes. The intermediate wavelength tested here showed a comparatively balanced response and was accompanied by enhanced IAA-related metabolism. Future studies should further elucidate the interfacial electron-transfer mechanisms, extend wavelength screening, and distinguish photochemical effects from bacterial metabolic responses. Collectively, this study establishes wavelength control as a tunable strategy for programming extracellular metabolism in bacteria–semiconductor biohybrids, expanding microbial semiconductor research from light-driven bioconversion toward wavelength-programmable regulation of microbial functions.

## Data Availability

The sequence data generated in this study have been deposited in GenBank under accession number PV688044. Other data supporting the findings of this study are available from the corresponding author upon reasonable request.

## Supplementary Materials

The following supporting information can be downloaded at: https://www.mdpi.com/article/10.3390/microorganisms14102218/s1, Figure S1: Size distribution of CdS nanoparticles synthesized in situ by *Priestia* ZJT-3; Figure S2: Size distribution of extracted CdS nanoparticles biomineralized by *Priestia* ZJT-3; Figure S3: PLS-DA score plot of extracellular metabolite profiles from all samples; Figure S4: Correlation heatmap showing the relationships among all samples; Figure S5: PLS-DA score plot showing the separation of groups based on differential metabolite profiles; Figure S6: Volcano plot of differential extracellular metabolites between 400 nm-illuminated and dark-treated *Priestia* ZJT-3-CdS biohybrids; Figure S7: Volcano plot of differential extracellular metabolites between 450 nm-illuminated and dark-treated *Priestia* ZJT-3-CdS biohybrids; Figure S8: Volcano plot of differential extracellular metabolites between 520 nm-illuminated and dark-treated *Priestia* ZJT-3-CdS biohybrids; Figure S9: VIP score plot of the top 30 differential extracellular metabolites in *Priestia* ZJT-3-CdS biohybrids under 400 nm illumination compared with dark conditions; Figure S10: Changes in bacterial density of the biohybrid system after 16 h of illumination at different wavelengths (initial OD_600_ = 1.0); Figure S11: SEM morphological changes in bacterial surface morphology of the biohybrid system after 16 h of illumination at different wavelengths; Figure S12: Changes in the cadmium concentration in the supernatant of the biohybrid system after 16 h of illumination at different wavelengths; Figure S13: VIP score plot of the top 30 differential extracellular metabolites in *Priestia* ZJT-3-CdS biohybrids under 450 nm illumination compared with dark conditions; Figure S14: VIP score plot of the top 30 differential extracellular metabolites in *Priestia* ZJT-3-CdS biohybrids under 520 nm illumination compared with dark conditions; Figure S15: KEGG pathway enrichment analysis of differential extracellular metabolites between 400 nm-illuminated and dark-treated *Priestia* ZJT-3-CdS biohybrids; Figure S16: KEGG pathway enrichment analysis of differential extracellular metabolites between 520 nm-illuminated and dark-treated *Priestia* ZJT-3-CdS biohybrids; Figure S17: VIP scores of 33 tryptophan metabolism-related extracellular metabolites between 400 nm-illuminated and dark-treated *Priestia* ZJT-3-CdS biohybrids; Figure S18: VIP scores of 33 tryptophan metabolism-related extracellular metabolites between 450 nm-illuminated and dark-treated *Priestia* ZJT-3-CdS biohybrids; Figure S19: VIP scores of 33 tryptophan metabolism-related extracellular metabolites between 520 nm-illuminated and dark-treated *Priestia* ZJT-3-CdS biohybrids.
