# Supplementary material for: Wavelength-Specific Reprogramming of the Extracellular Metabolome in Bacterial CdS Biohybrids
Source: Microorganisms. 2026 Oct 2;14(10):2218. doi: 10.3390/microorganisms14102218 (PMC13707940; doi:10.3390/microorganisms14102218)
Supplement: Supplementary file 1 [file microorganisms-14-02218-s001.zip › microorganisms-4564973-supplementary.pdf]

Supporting Information

Wavelength-Specific Reprogramming of the Extracellular Metabolome in Bacterial CdS Biohybrids

Tan Ke [1,*], Xiaojie Ni [1], Liang Guo [1], Kai Zheng [1], Keren Lu [1] and Yue Tao [2]

[1] Zhejiang international Joint Laboratory on Low-Carbon Pollution Control and Resource Utilization, School of Biological and Environmental Engineering, Zhejiang Shuren University, Hangzhou 310015, China

[2] State Key Laboratory of Vegetation Structure, Function and Construction (VegLab), Institute of Ecology, College of Urban and Environmental Sciences, Peking University, Beijing 100080, China

* Correspondence: ketan94@zjsru.edu.cn

List of Supporting information

Figure S1. Size distribution of CdS nanoparticles synthesized in situ by *Priestia* ZJT-3.

Figure S2. Size distribution of extracted CdS nanoparticles biomineralized by *Priestia* ZJT-3.

Figure S3. PLS-DA score plot of extracellular metabolite profiles from all samples.

Figure S4. Correlation heatmap showing the relationships among all samples.

Figure S5. PLS-DA score plot showing the separation of groups based on differential metabolite profiles.

Figure S6. Volcano plot of differential extracellular metabolites between 400 nm-illuminated and dark-treated *Priestia* ZJT-3-CdS biohybrids.

Figure S7. Volcano plot of differential extracellular metabolites between 450 nm-illuminated and dark-treated *Priestia* ZJT-3-CdS biohybrids.

Figure S8. Volcano plot of differential extracellular metabolites between 520 nm-illuminated and dark-treated *Priestia* ZJT-3-CdS biohybrids.

Figure S9. VIP score plot of the top 30 differential extracellular metabolites in *Priestia* ZJT-3-CdS biohybrids under 400 nm illumination compared with dark conditions.

Figure S10. Changes in bacterial density of the biohybrid system after 16 h of illumination at different wavelengths (initial OD_{600} = 1.0).

Figure S11. SEM morphological changes in bacterial surface morphology of the biohybrid system after 16 h of illumination at different wavelengths.

Figure S12. Changes in the cadmium concentration in the supernatant of the biohybrid system after 16 h of illumination at different wavelengths.

Figure S13. VIP score plot of the top 30 differential extracellular metabolites in *Priestia* ZJT-3-CdS biohybrids under 450 nm illumination compared with dark conditions..

Figure S14. VIP score plot of the top 30 differential extracellular metabolites in *Priestia* ZJT-3-CdS biohybrids under 520 nm illumination compared with dark conditions.

Figure S15. KEGG pathway enrichment analysis of differential extracellular metabolites between 400 nm-illuminated and dark-treated *Priestia* ZJT-3-CdS biohybrids.

Figure S16. KEGG pathway enrichment analysis of differential extracellular metabolites between 520 nm-illuminated and dark-treated *Priestia* ZJT-3-CdS biohybrids.

Figure S17. VIP scores of 33 tryptophan metabolism-related extracellular metabolites between 400 nm-illuminated and dark-treated *Priestia* ZJT-3-CdS biohybrids.

Figure S18. VIP scores of 33 tryptophan metabolism-related extracellular metabolites between 450 nm-illuminated and dark-treated *Priestia* ZJT-3-CdS biohybrids.

Figure S19. VIP scores of 33 tryptophan metabolism-related extracellular metabolites between 520 nm-illuminated and dark-treated *Priestia* ZJT-3-CdS biohybrids.

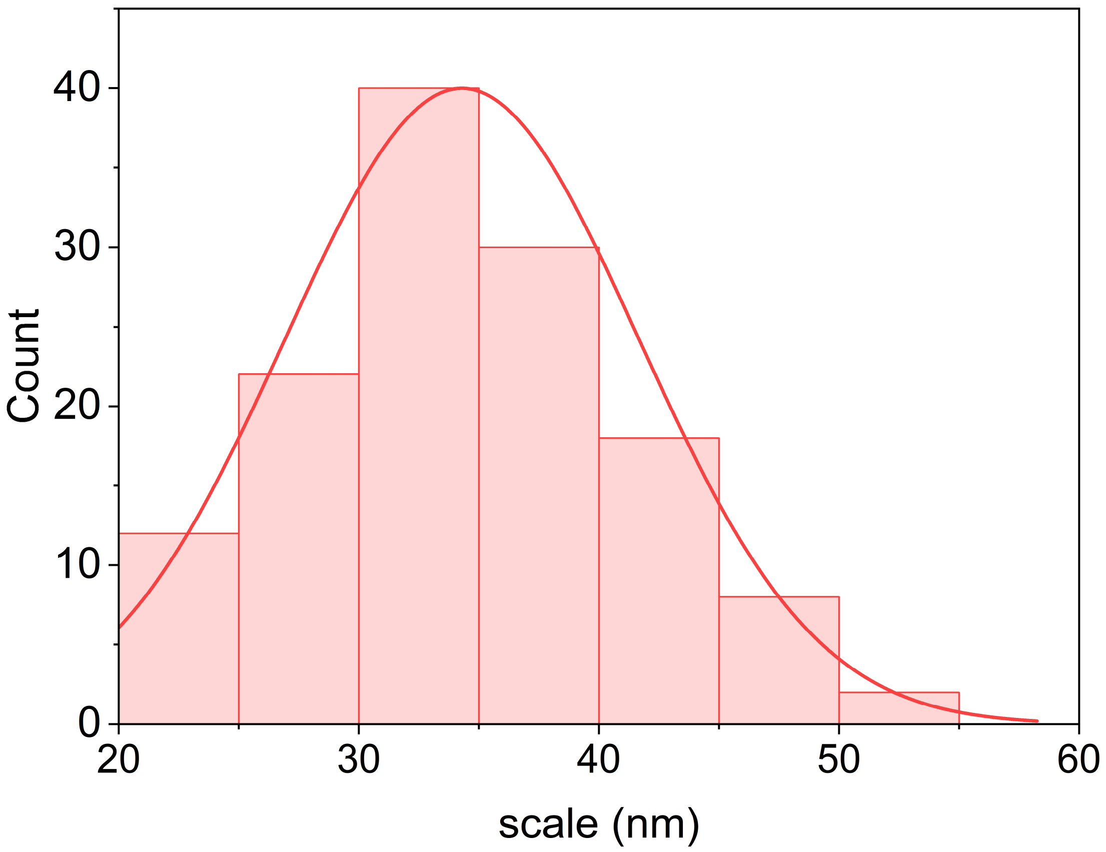

Figure S1. Size distribution of CdS nanoparticles synthesized in situ by *Priestia* ZJT-3.

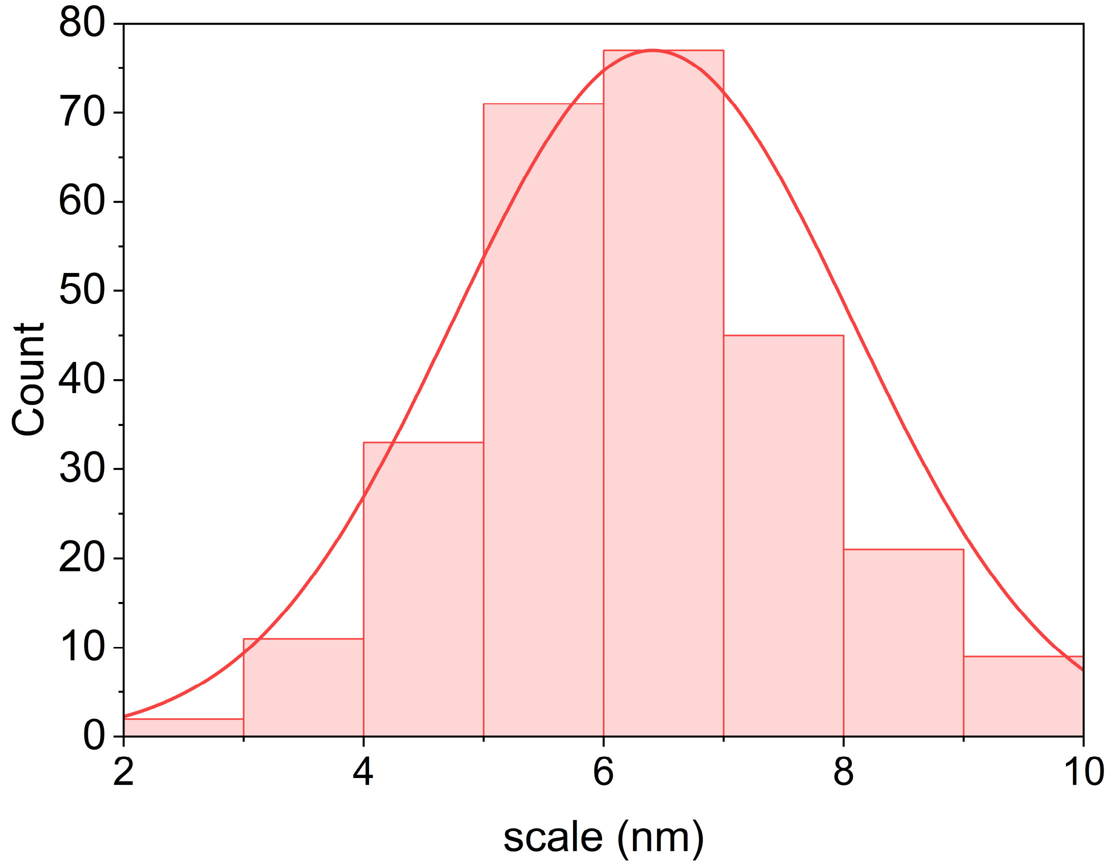

Figure S2. Size distribution of extracted CdS nanoparticles biomineralized by *Priestia* ZJT-3.

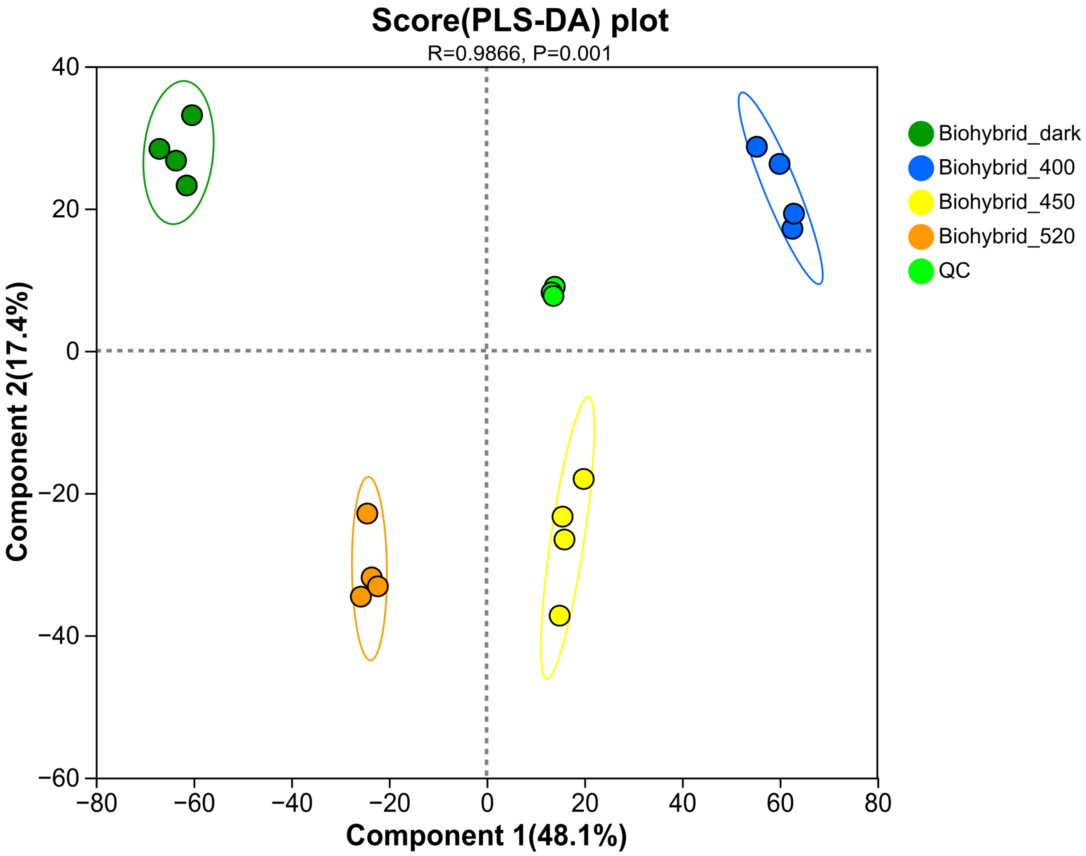

Figure S3. PLS-DA score plot of extracellular metabolite profiles from all samples.

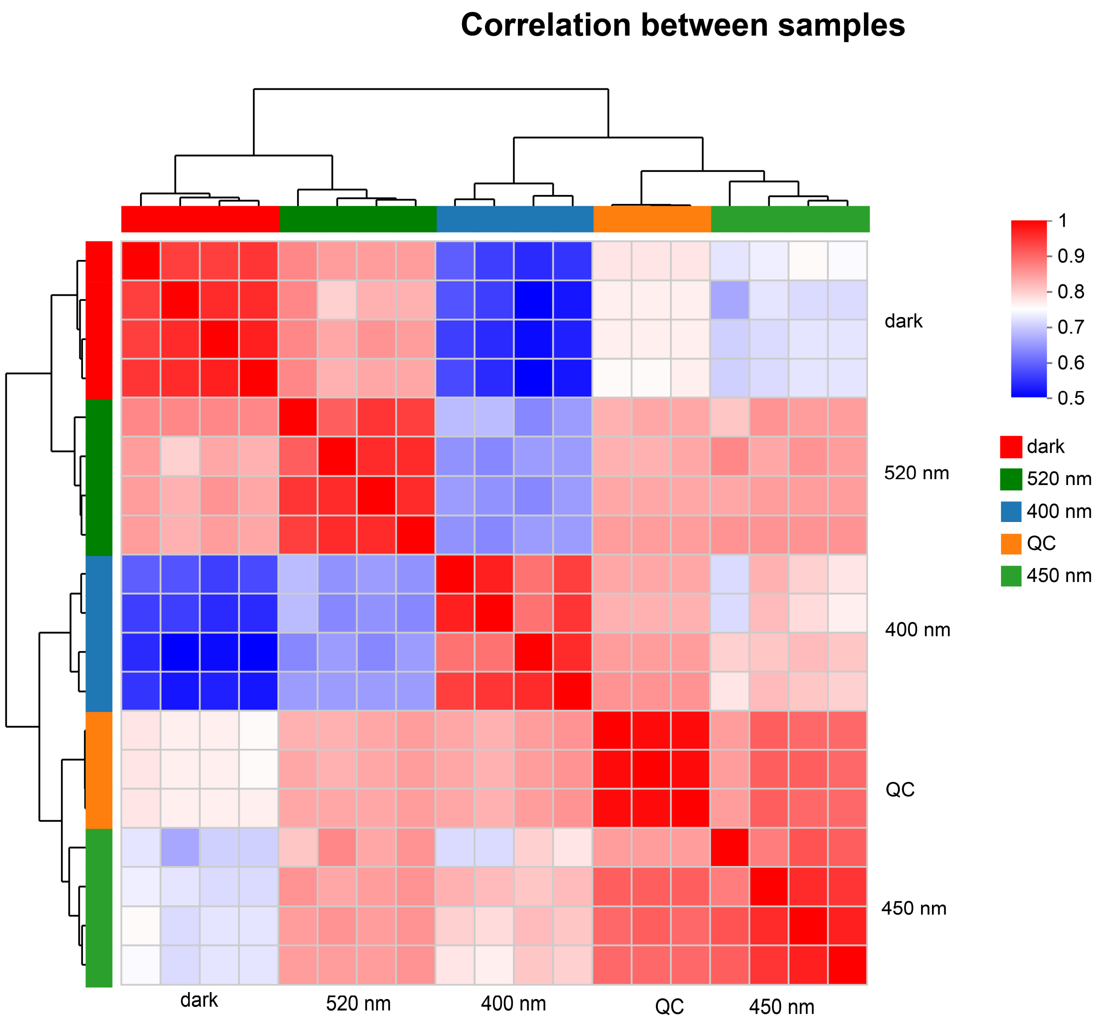

Figure S4. Correlation heatmap showing the relationships among all samples.

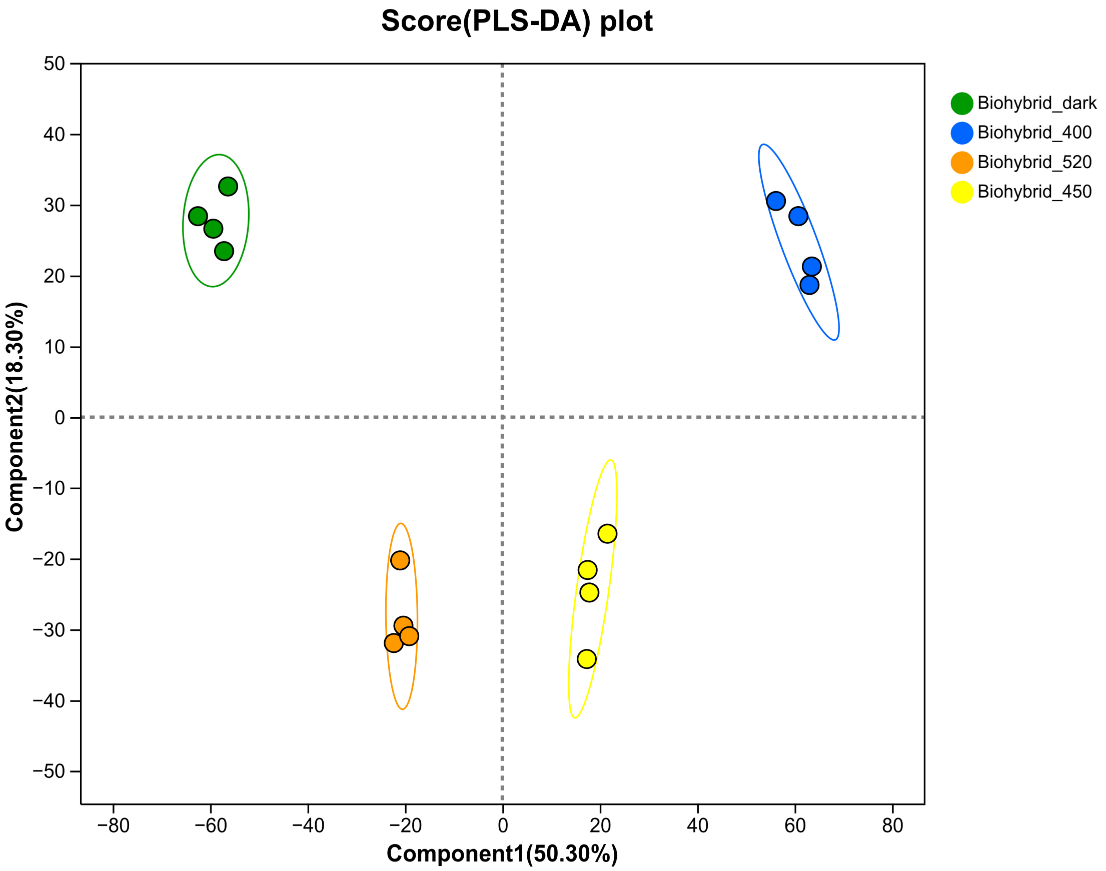

Figure S5. PLS-DA score plot showing the separation of groups based on differential metabolite profiles.

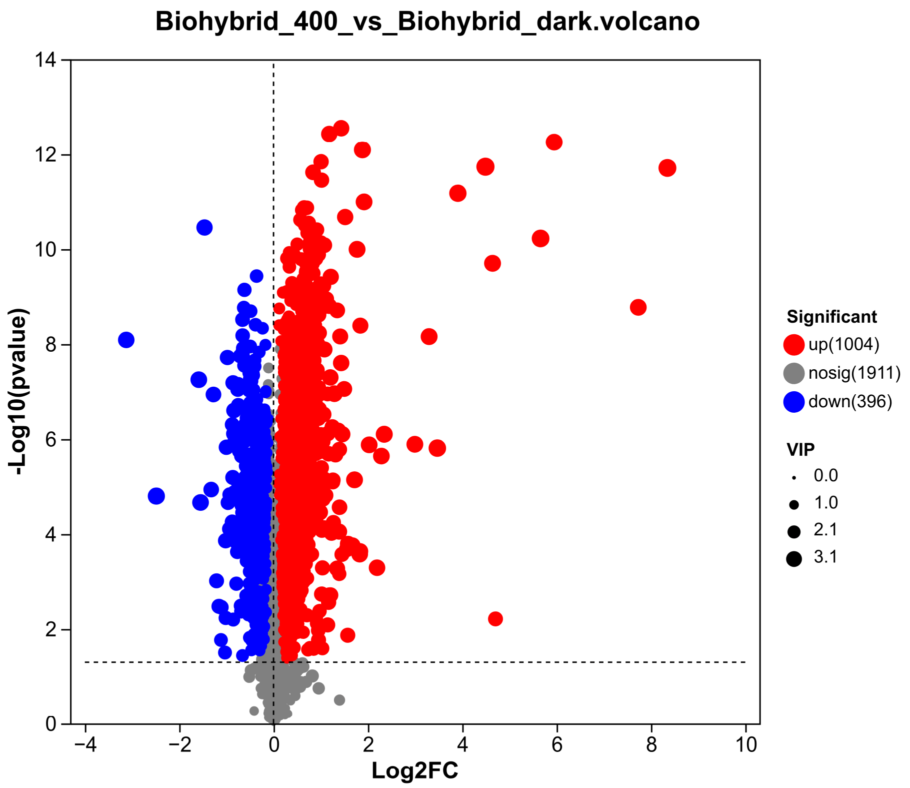

Figure S6. Volcano plot of differential extracellular metabolites between 400 nm-illuminated and dark-treated *Priestia* ZJT-3-CdS biohybrids.

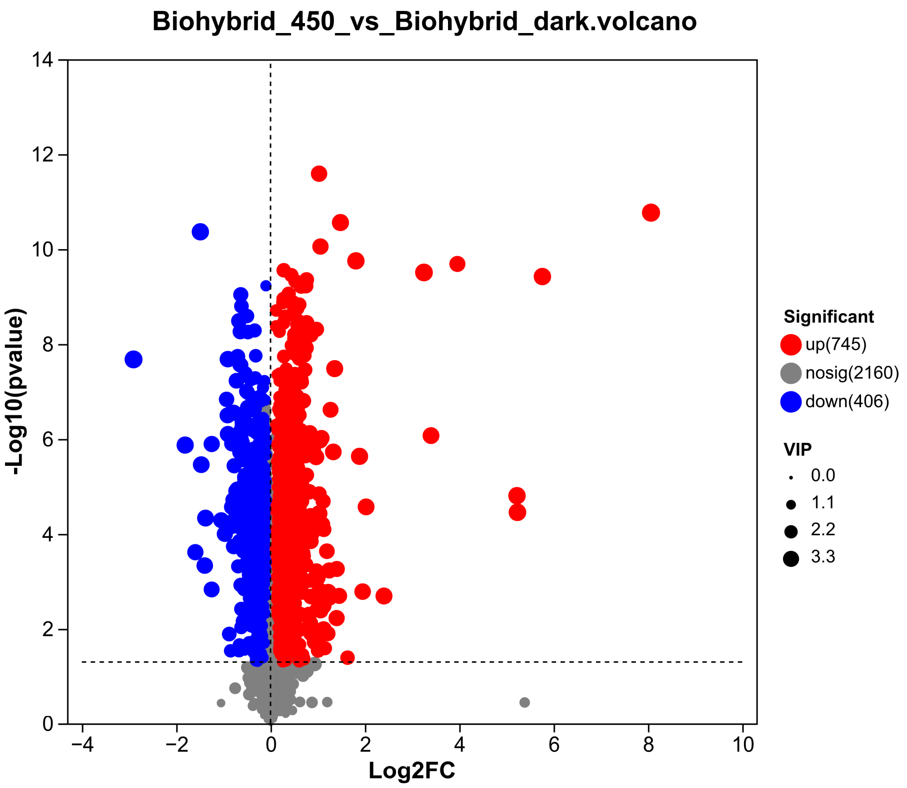

Figure S7. Volcano plot of differential extracellular metabolites between 450 nm-illuminated and dark-treated *Priestia* ZJT-3-CdS biohybrids.

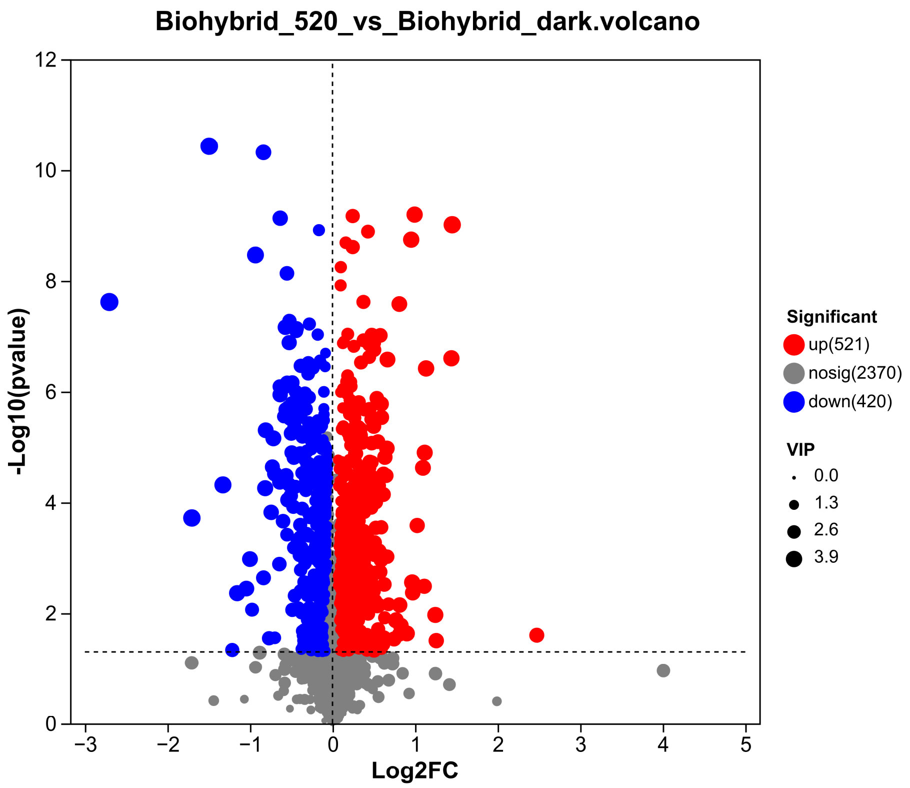

Figure S8. Volcano plot of differential extracellular metabolites between 520 nm-illuminated and dark-treated *Priestia* ZJT-3-CdS biohybrids.

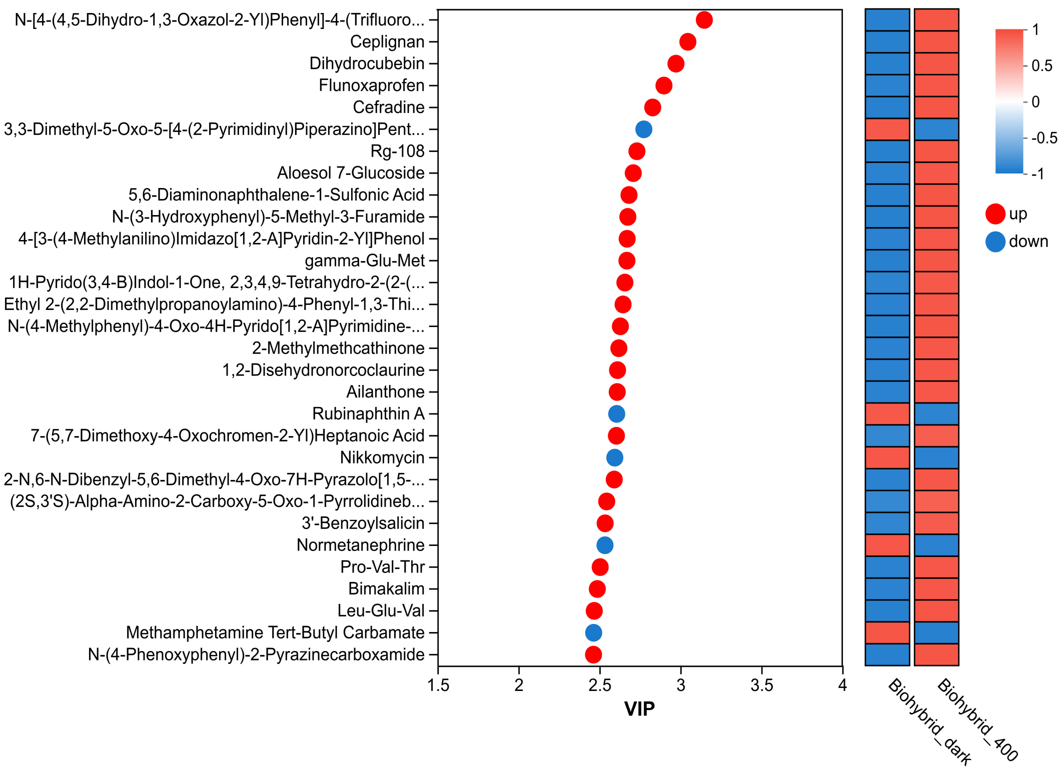

Figure S9. VIP score plot of the top 30 differential extracellular metabolites in *Priestia* ZJT-3-CdS biohybrids under 400 nm illumination compared with dark conditions.

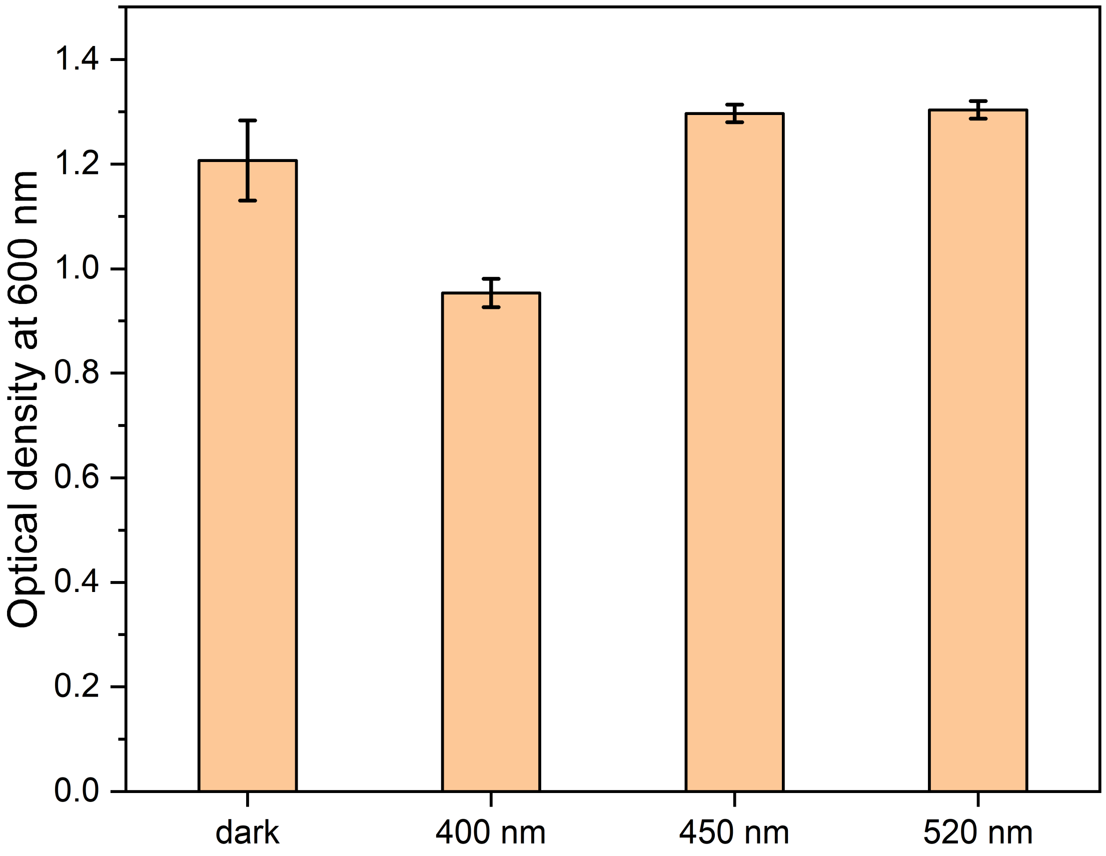

Figure S10. Changes in bacterial density of the biohybrid system after 16 h of illumination at different wavelengths (initial OD_{600} = 1.0).

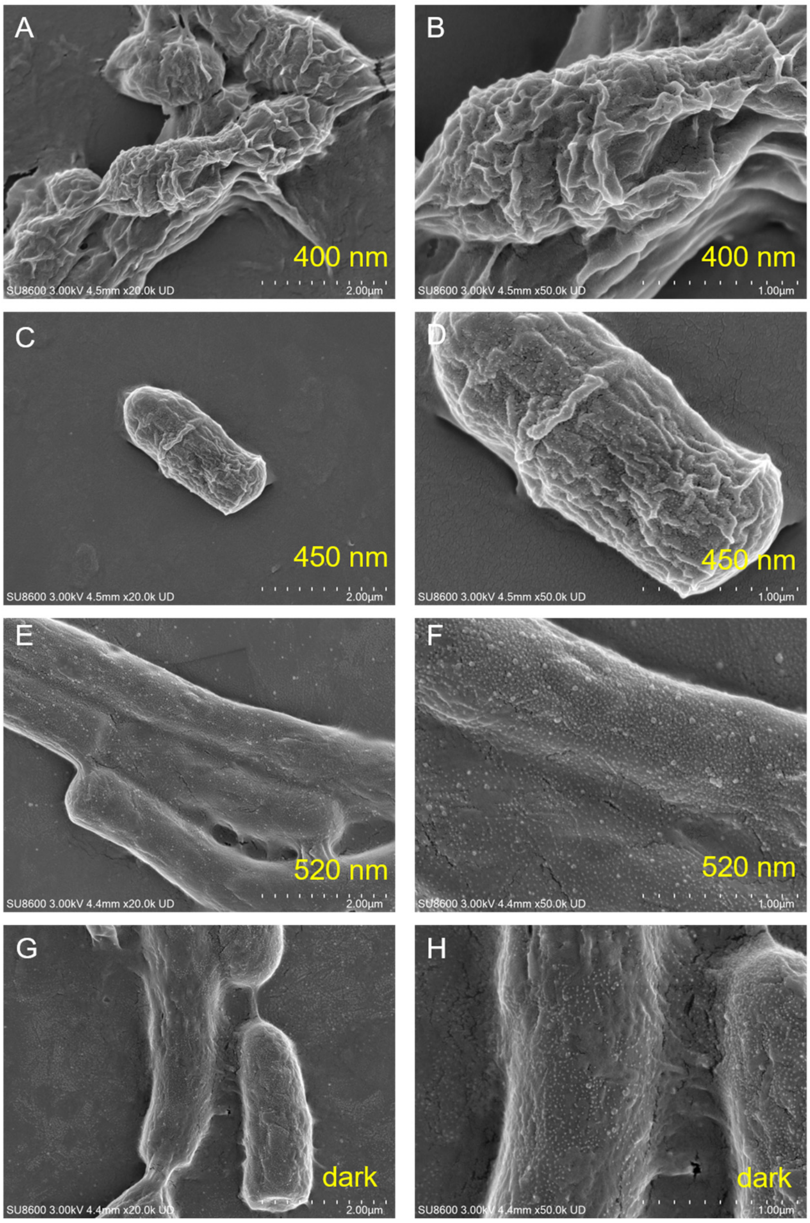

Figure S11. SEM morphological changes in bacterial surface morphology of the biohybrid system after 16 h of illumination at different wavelengths.

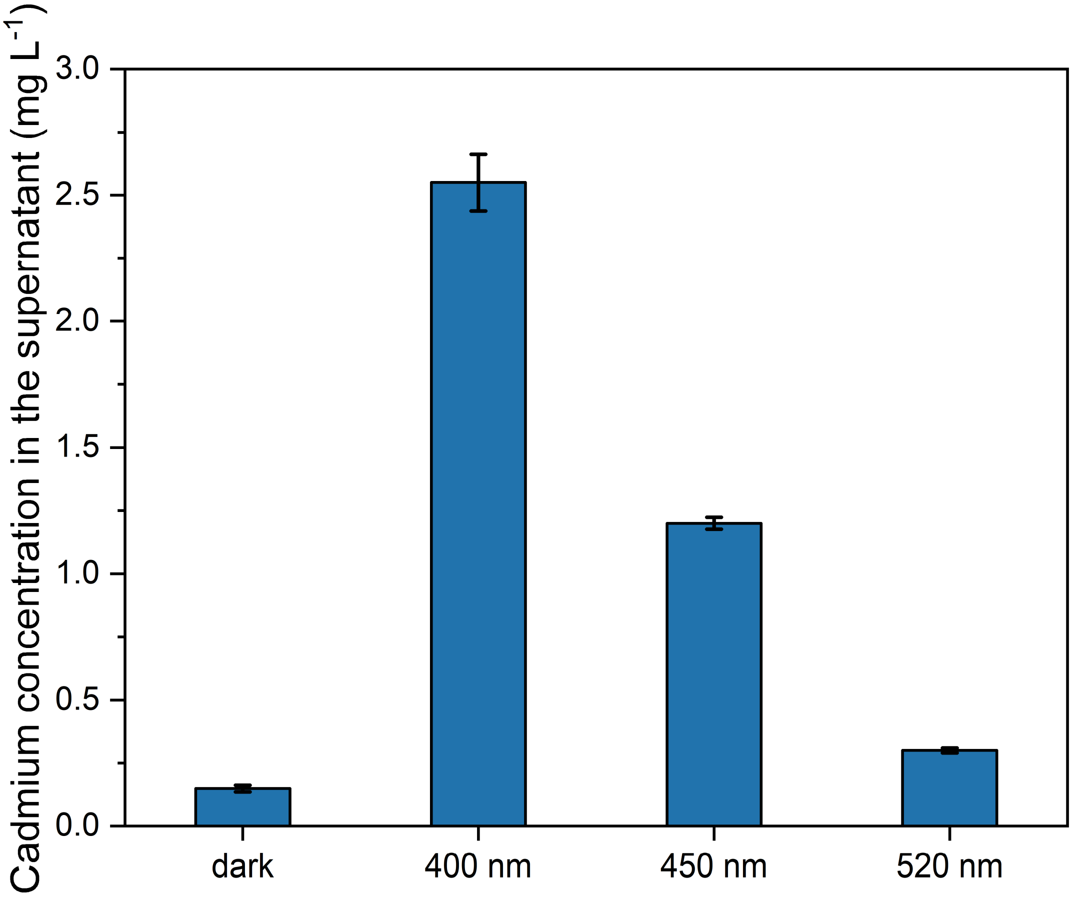

Figure S12. Changes in the cadmium concentration in the supernatant of the biohybrid system after 16 h of illumination at different wavelengths.

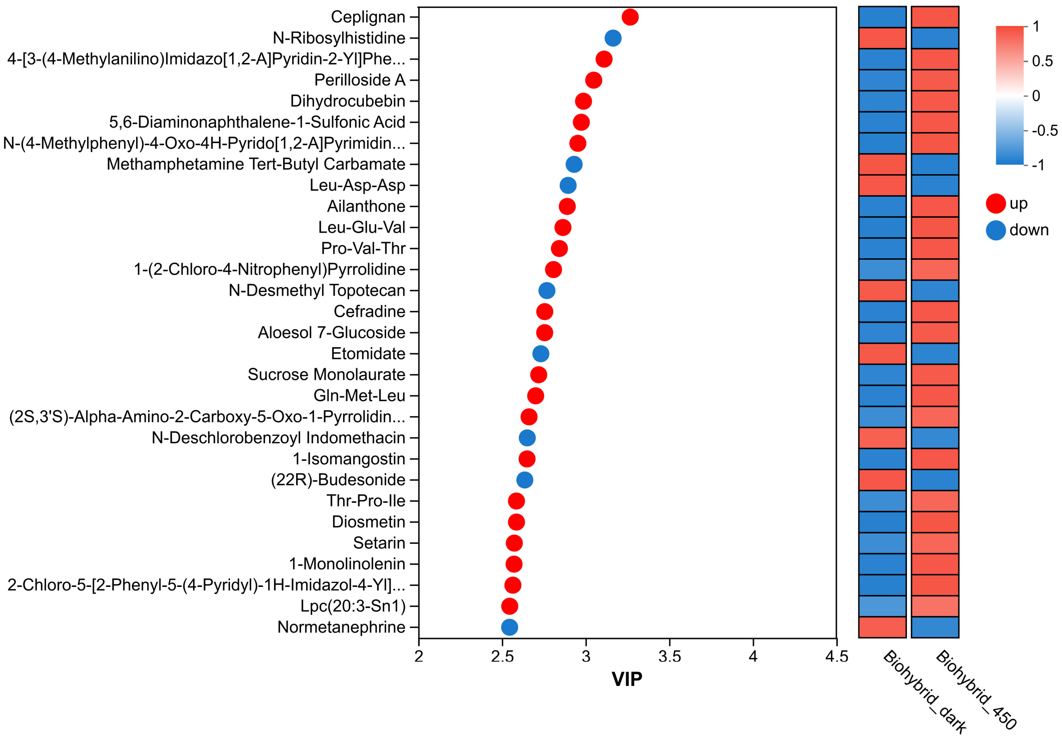

Figure S13. VIP score plot of the top 30 differential extracellular metabolites in *Priestia* ZJT-3-CdS biohybrids under 450 nm illumination compared with dark conditions.

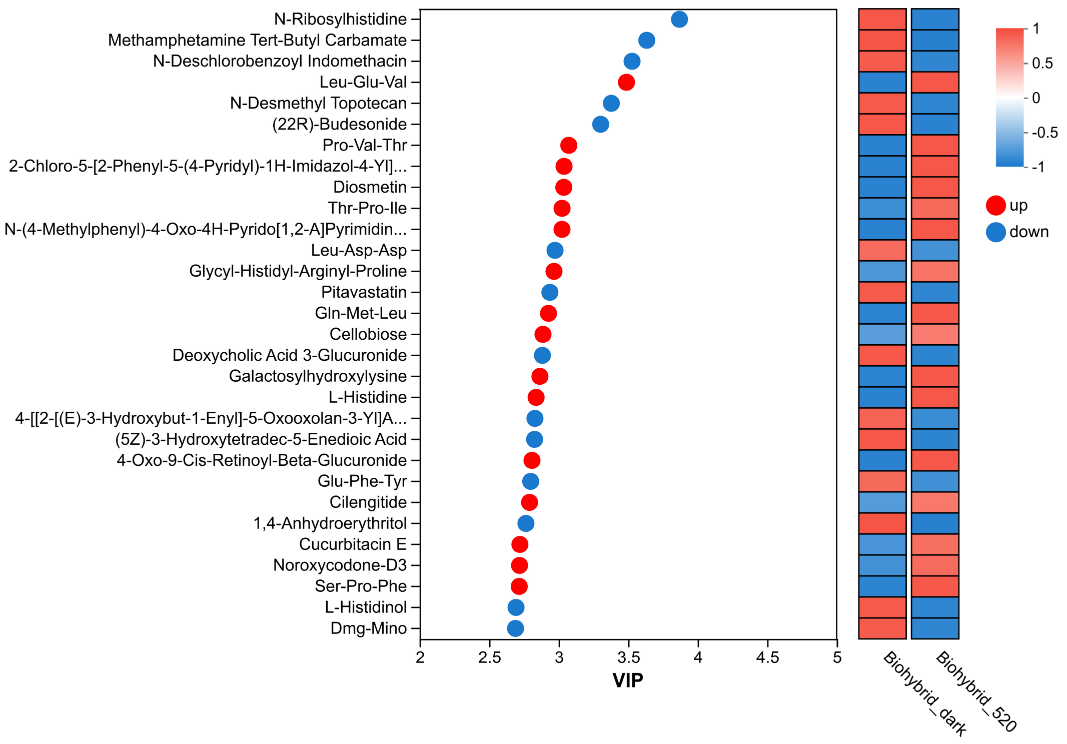

Figure S14. VIP score plot of the top 30 differential extracellular metabolites in *Priestia* ZJT-3-CdS biohybrids under 520 nm illumination compared with dark conditions.

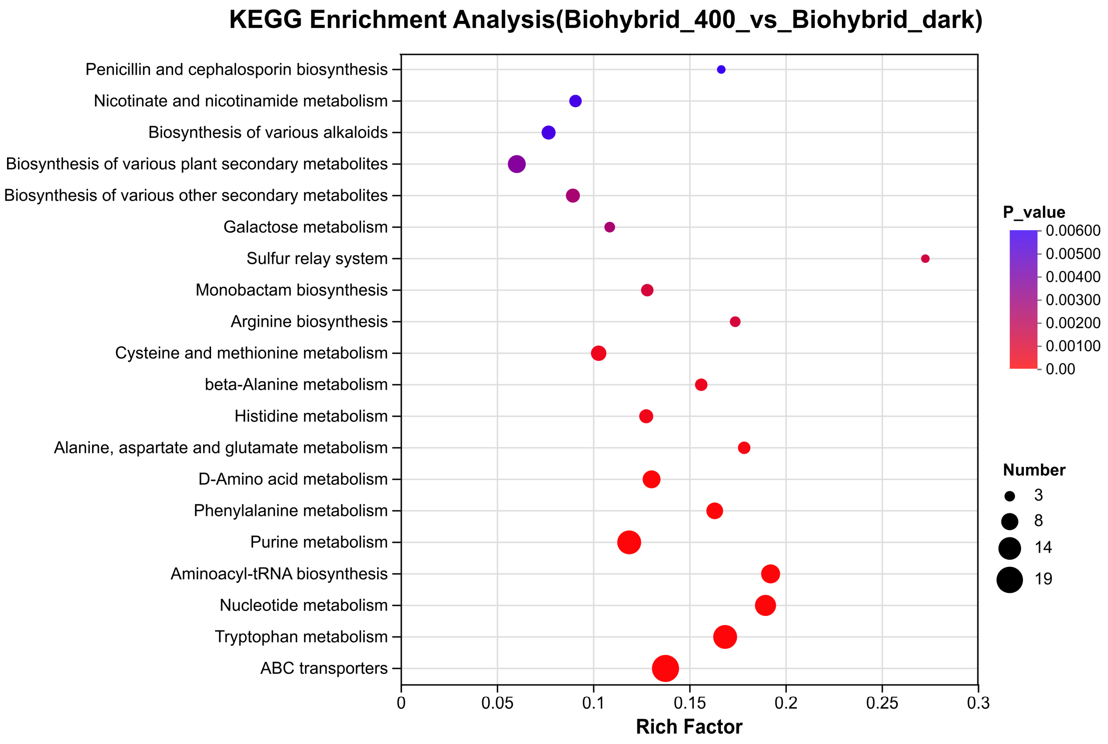

Figure S15. KEGG pathway enrichment analysis of differential extracellular metabolites between 400 nm-illuminated and dark-treated *Priestia* ZJT-3-CdS biohybrids.

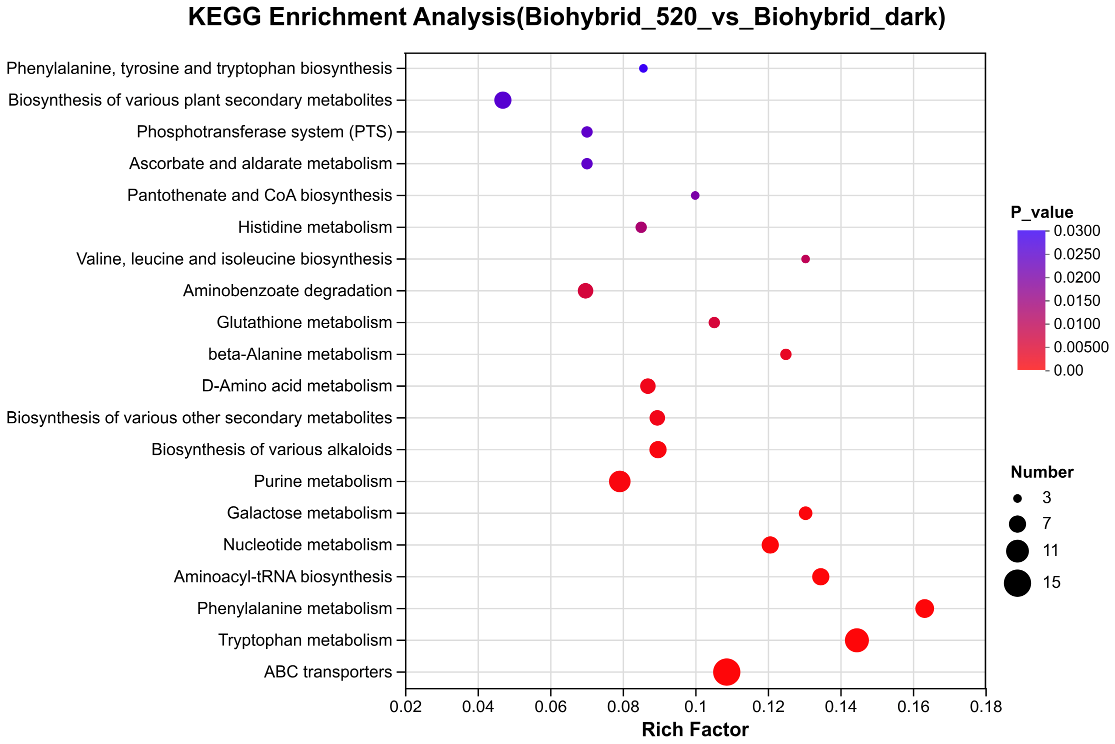

Figure S16. KEGG pathway enrichment analysis of differential extracellular metabolites between 520 nm-illuminated and dark-treated *Priestia* ZJT-3-CdS biohybrids.

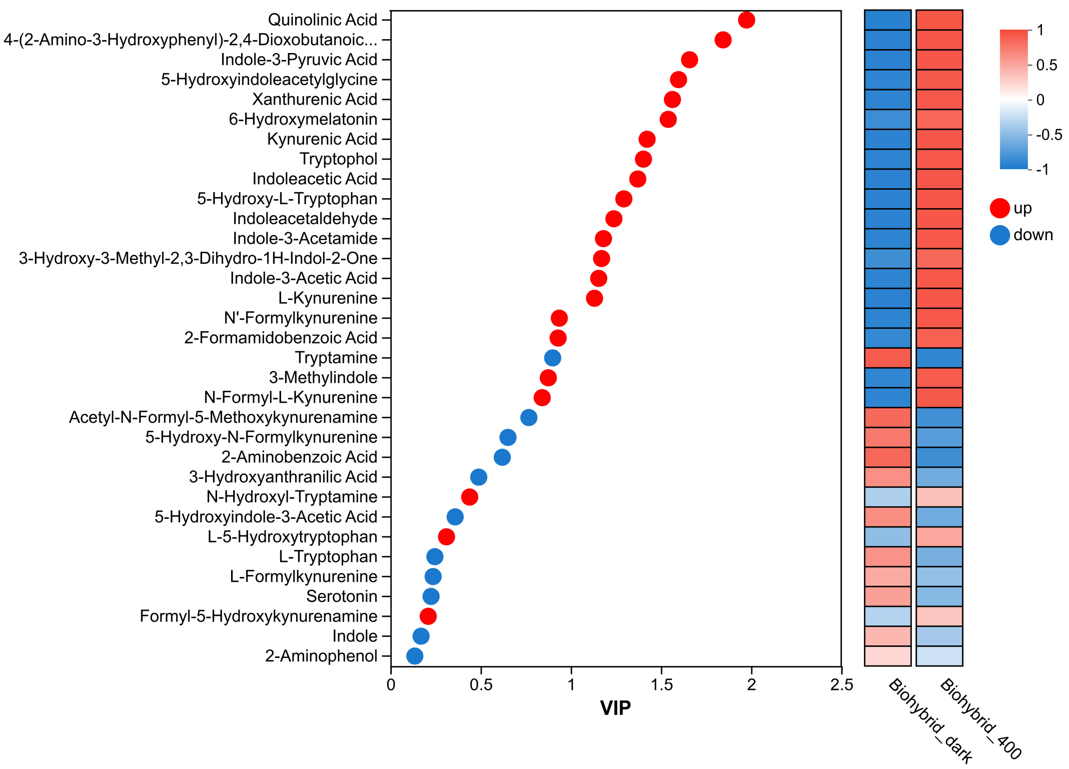

Figure S17. VIP scores of 33 tryptophan metabolism-related extracellular metabolites between 400 nm-illuminated and dark-treated *Priestia* ZJT-3-CdS biohybrids.

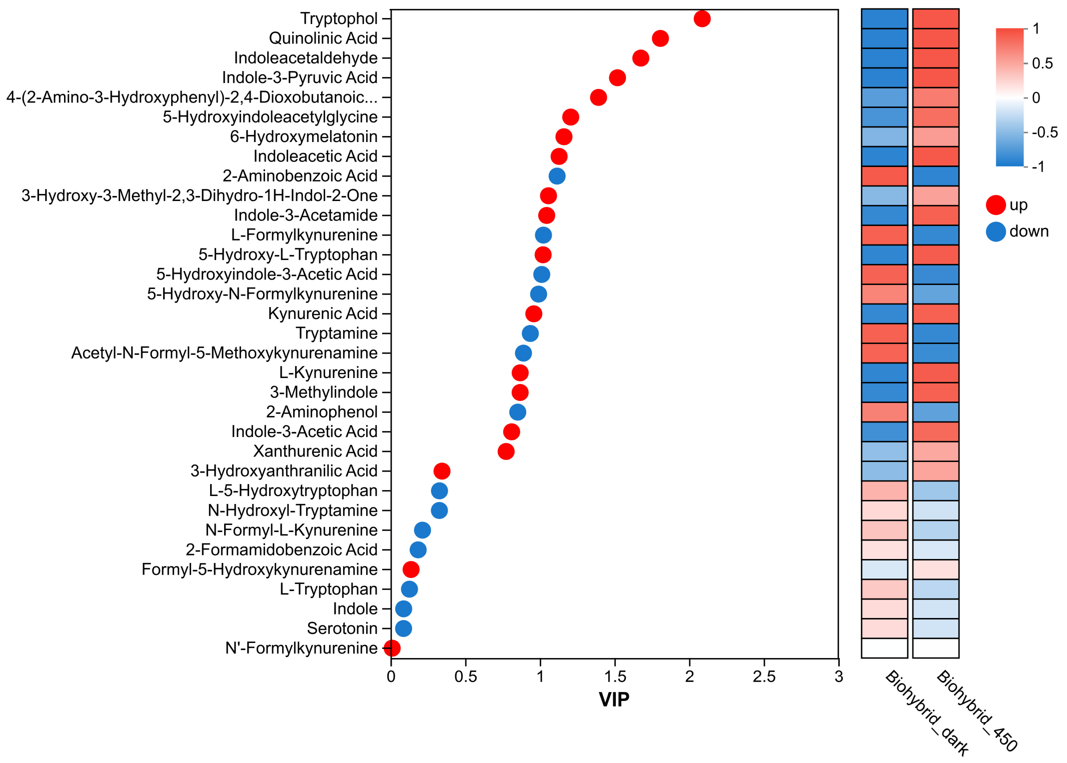

Figure S18. VIP scores of 33 tryptophan metabolism-related extracellular metabolites between 450 nm-illuminated and dark-treated *Priestia* ZJT-3-CdS biohybrids.

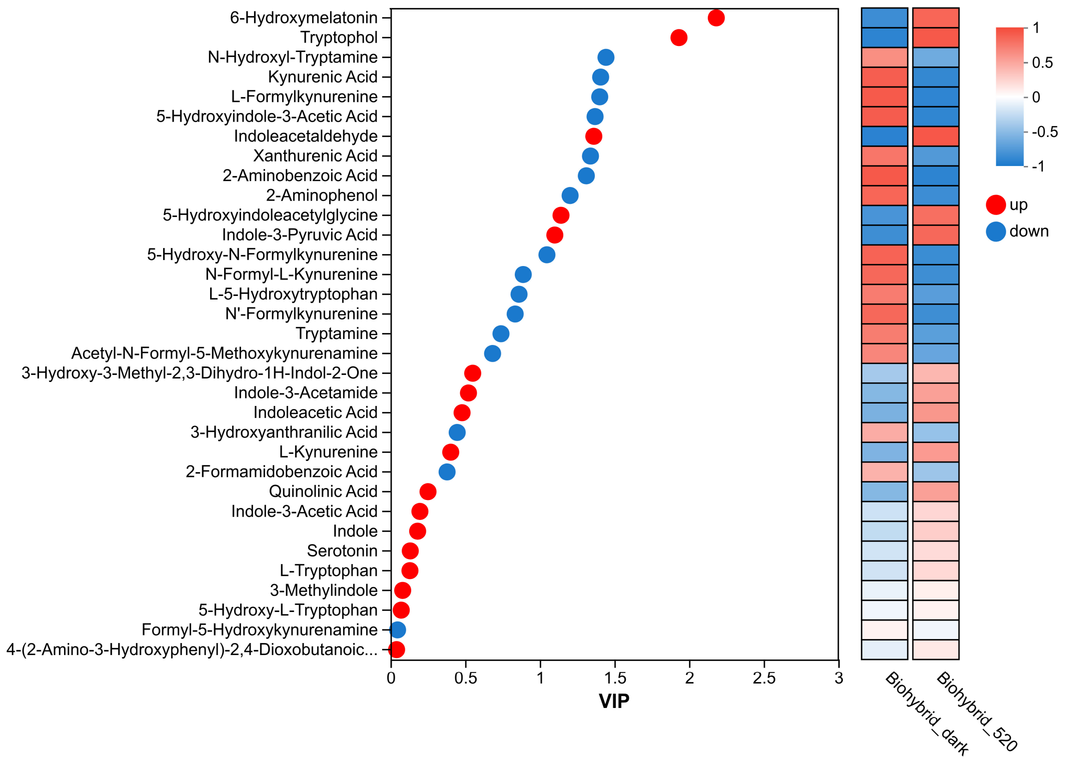

Figure S19. VIP scores of 33 tryptophan metabolism-related extracellular metabolites between 520 nm-illuminated and dark-treated *Priestia* ZJT-3-CdS biohybrids.
